# Robust hepatitis E virus infection and transcriptional response in human hepatocytes

**DOI:** 10.1073/pnas.1912307117

**Published:** 2020-01-02

**Authors:** Daniel Todt, Martina Friesland, Nora Moeller, Dimas Praditya, Volker Kinast, Yannick Brüggemann, Leonard Knegendorf, Thomas Burkard, Joerg Steinmann, Rani Burm, Lieven Verhoye, Avista Wahid, Toni Luise Meister, Michael Engelmann, Vanessa M. Pfankuche, Christina Puff, Florian W. R. Vondran, Wolfgang Baumgärtner, Philip Meuleman, Patrick Behrendt, Eike Steinmann

**Affiliations:** ^a^Department of Molecular and Medical Virology, Ruhr University Bochum, 44801 Bochum, Germany;; ^b^Institute for Experimental Virology, TWINCORE Centre for Experimental and Clinical Infection Research, a Joint Venture between the Medical School Hannover (MHH) and the Helmholtz Centre for Infection Research (HZI), 30625 Hannover, Germany;; ^c^European Virus Bioinformatics Center (EVBC), 07743 Jena, Germany;; ^d^Institute of Medical Microbiology, University Hospital of Essen, 45147 Essen, Germany;; ^e^Institute of Clinical Hygiene, Medical Microbiology and Infection, Paracelsus Medical University, 90419 Nürnberg, Germany;; ^f^Laboratory of Liver Infectious Diseases, Department of Diagnostic Sciences, Faculty of Medicine and Health Sciences, Ghent University, 9000 Ghent, Belgium;; ^g^Department of Pathology, University of Veterinary Medicine Hannover, 30559 Hannover, Germany;; ^h^Regenerative Medicine and Experimental Surgery (ReMediES), Department of General, Visceral and Transplantation Surgery, Hannover Medical School, 30625 Hannover, Germany;; ^i^German Centre for Infection Research (DZIF), Hannover-Braunschweig, 30625 Hannover, Germany;; ^j^Department of Gastroenterology, Hepatology and Endocrinology, Hannover Medical School, 30625 Hannover, Germany

**Keywords:** hepatitis E virus (HEV), infection, primary hepatocytes, humanized mice, transcriptomics

## Abstract

Hepatitis E virus (HEV) is the causative agent of hepatitis E in humans and the leading cause for acute viral hepatitis worldwide. The virus is classified as a member of the genus *Orthohepevirus A* within the *Hepeviridae* family. Due to the absence of a robust cell culture model for HEV infection, the analysis of the viral life cycle, the development of effective antivirals and a vaccine is severely limited. In this study, we established a protocol based on the HEV genotype 3 p6 (Kernow C-1) and the human hepatoma cell lines HepG2 and HepG2/C3A with different media conditions to produce intracellular HEV cell culture-derived particles (HEVcc) with viral titers between 10^5^ and 10^6^ FFU/mL. Viral titers could be further enhanced by an HEV variant harboring a mutation in the RNA-dependent RNA polymerase. These HEVcc particles were characterized in density gradients and allowed the *trans*-complementation of subgenomic reporter HEV replicons. In addition, in vitro produced intracellular-derived particles were infectious in liver-humanized mice with high RNA copy numbers detectable in serum and feces. Efficient infection of primary human and swine hepatocytes using the developed protocol could be observed and was inhibited by ribavirin. Finally, RNA sequencing studies of HEV-infected primary human hepatocytes demonstrated a temporally structured transcriptional defense response. In conclusion, this robust cell culture model of HEV infection provides a powerful tool for studying viral–host interactions that should facilitate the discovery of antiviral drugs for this important zoonotic pathogen.

## Significance

Chronic HEV infections pose a significant clinical problem in immunocompromised individuals. The lack of an efficient cell culture system has severely limited investigation of the HEV life cycle and the development of effective antivirals. Here we report the establishment of a robust HEV cell culture system in human hepatocytes with viral titers up to 10^6^ FFU/mL. These produced intracellular-derived HEVcc particles demonstrated replication to high viral loads in human liver chimeric mice and were able to efficiently infect primary human as well as porcine hepatocytes. This unique infectious cell culture model provides a powerful tool for the analysis of host–virus interactions that should facilitate the discovery of antiviral drugs for this important zoonotic pathogen.

Hepatitis E virus (HEV) is a positive-orientated, single-stranded RNA virus and the causative agent of hepatitis E in humans. The virus is classified as a member of the genus *Orthohepevirus A* within the *Hepeviridae* family. With more than 20 million infections per year, it is responsible for the majority of acute hepatitis worldwide leading to up to 70,000 deaths (1). At least 4 human-pathogenic HEV genoytpes have been described (gt 1 to 4). Genotype 1 and 2 solely infect humans and are mainly present in developing areas causing periodically waterborne outbreaks via the fecal–oral infection pathway (2). Especially pregnant women harbor a high risk for a fatal outcome during HEV gt 1 infection with mortality rates up to 30% in the last trimester (3). In contrast, gt 3 and 4 are zoonotic pathogens with their main reservoir in pigs, wild boars, and deer (4). Therefore, major risk factors for virus transmission include contact with these animals or consumption of contaminated meat products. The latter genotypes are responsible for most of the infections in developed nations. HEV gt 3 infections in humans are usually self-limiting. However, in patients with preexisting liver disease, acute-on-chronic liver failure can develop. Additionally, HEV gt 3 infections can progress also to a chronic stage in immunosuppressed individuals with the risk for the rapid development of liver cirrhosis and eventually hepatic decompensation with the need for liver transplantation (5). There is no recommended specific treatment for patients with acute-on-chronic liver failure caused by HEV. The current therapeutic options are limited to the off-label use of ribavirin (RBV) and pegylated IFN-α (pegIFN-α), which are often associated with severe side effects and are contraindicated in pregnant women (6, 7).

HEV is a quasi-enveloped virus circulating in the nonenveloped state in bile and feces but is found wrapped into cellular membranes in the blood stream (8). The 7.2-kb RNA capped genome encodes for 3 ORFs: the nonstructural polyprotein required for RNA replication (ORF1), the capsid protein of the HEV virion (ORF2), and a small multifunctional protein with a molecular mass of 13 kDa (ORF3) (9). The HEV life cycle and host–virus interactions that determine the outcome of infection have been difficult to study, especially because robust cell culture models for HEV were not available in the past. This long absence of in vitro systems also severely limited the development of effective antivirals and vaccines targeting HEV. Many different cell culture systems have been tested in the past using various HEV strains, but mostly viral replication progresses very slowly and infection with low virion counts often results in nonproductive infection (10–12). Recent breakthroughs have been achieved by identifying compatible cell lines and specific HEV strains (11). In this study, we report the establishment of a robust HEV cell culture system based on an HEV gt 3 recombinant cDNA clone and the human hepatoma cell lines HepG2 and HepG2/C3A to produce intracellular HEV cell culture-derived particles (HEVcc) with viral titers up to 10^6^ focus forming units (FFU)/mL. We observed efficient infection of primary human and swine hepatocytes as well as in vivo propagation with high viral loads in liver-humanized mice. Furthermore, study of dynamic viral–host interactions via transcriptomic network analysis after HEV infection of primary human hepatocytes (PHH) revealed distinct temporal antiviral responses.

## Materials and Methods

### HEV Constructs and in Vitro Transcription.

A plasmid construct containing the full-length HEV genome (Kernow-C1 p6 clone, gt3; GenBank accession no. JQ679013) and a variant harboring an RNA-dependent RNA polymerase mutation G1634R (13) were used to generate HEV in vitro transcripts as previously described (14). Capping of the constructs was performed using Ribo m7G Cap Analog (Promega). A subgenomic Kernow-C1 p6 HEV sequence coupled to a *Gaussia* luciferase reporter gene was used as described before (15). A HEV p6-based GFP reporter construct (green fluorescent protein) was constructed by replacing the *Gaussia* luciferase. A plasmid encoding the full-length HEV infectious clone HEV83-2-27 (GenBank accession no. AB740232) (16) and a respective *Gaussia* luciferase reporter replicon therefore were kindly provided by Koji Ishii as well as Takaji Wakita (Department of Virology II, National Institute of Infectious Diseases, Tokyo, Japan). Further details regarding the cloning strategies and exact nucleotide sequences can be obtained upon request.

### HEV Infectious Virus Production Assays.

For transfection we used the electroporation technique in accordance to previous reports (17). In brief, 9 × 10^6^ HepG2 or HepG2/C3A cells were resuspended in 400 µL Cytomix containing 2 mM ATP and 5 mM glutathione, mixed with 5 or 10 µg of HEV RNA and subsequently electroporated. Cells were immediately transferred to 13.6 mL of either DMEM complete or MEM low IgG FCS, and the cell suspension was seeded in respective plates (7.8 × 10^5^ to 1.3 × 10^6^ cells per well for 6-well plates, 7.1 × 10^6^ for 10-cm dishes, and 1.5 to 2.5 × 10^5^ cells per well for 24-well plates). After 24 h the medium was changed to fresh medium. Viral particle production was determined at designated time points posttransfection (p.t.) by harvesting the extracellular particles in the filtered (0.45 µm) supernatant and the intracellular virus by resuspension of the cells in a 5 times lower volume of the respective medium comparing to the harvested supernatant and lysis by 3 repeated freeze and thaw cycles. After a high-speed centrifugation step which separates the cell debris, the supernatant was harvested. Twenty-four-well plates were used for indirect immunofluorescence stain to check for HEV capsid-positive cells at designated time points

### Data Availability.

The RNAseq data discussed in this publication have been deposited in National Center for Biotechnology Information’s Gene Expression Omnibus (GEO) (18) and are accessible through GEO Series accession number GSE135619 (https://www.ncbi.nlm.nih.gov/geo/query/acc.cgi?acc=GSE135619).

All materials, data, and associated protocols will be made available upon request.

Additional materials and methods are posted in *SI Appendix*.

## Results

### Production of High-Titer HEVcc by a Combination of Different Hepatoma Cells and Media Conditions.

**Fig. 1.**
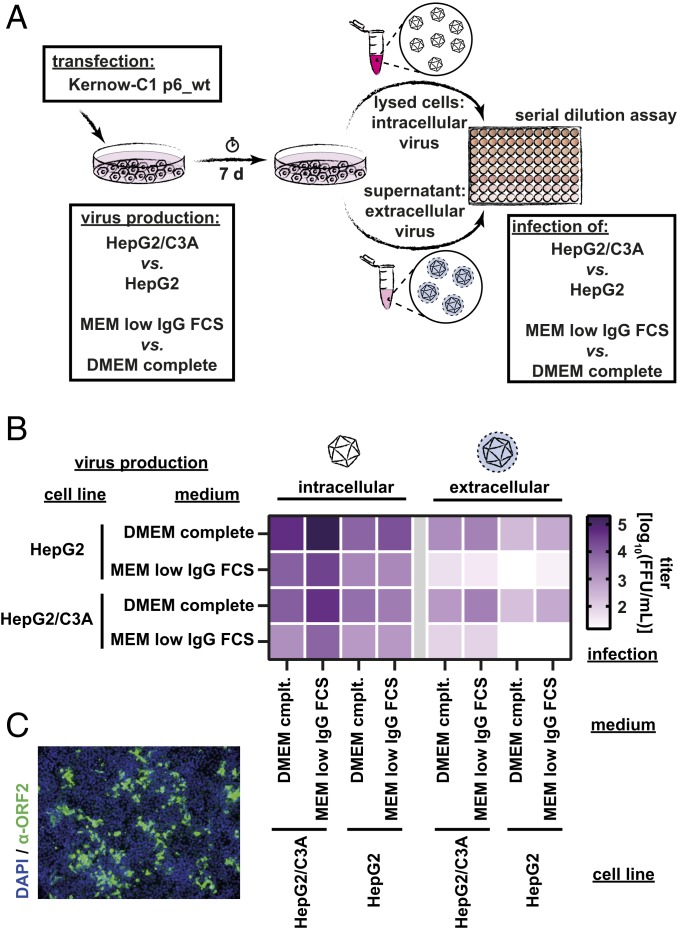
Optimizing cell culture conditions for high-titer virus production. (*A*) Workflow of HEV particle production and infection of target cells. HepG2 cells or a subclone thereof, HepG2/C3A cells, were transfected with in vitro transcribed HEV p6_wt RNA. Each cell line was cultivated in either DMEM complete or a medium with lowered IgG levels (MEM low IgG FCS). After 7 d, extracellular and intracellular virus particles, resembling the enveloped and nonenveloped states of HEV, were harvested and used to inoculate both naïve cell lines cultivated in the 2 media. In total, 32 different conditions were tested. (*B*) Heat map displaying mean titers of different combinations of cell lines and culture media used for the production of viral particles (rows, labeling to the left) and infection of target cells (columns, labeling below) for both intracellularly and extracellularly harvested particles. Viral titers are expressed as mean log of FFU/mL of 3 independent biological replicates (*n* = 3). (*C*) Immunofluorescence staining of HEV ORF2-positive foci under optimized conditions. Representative example after production of intracellular virus in HepG2 cells cultivated in DMEM complete and infection of HepG2/C3A cultivated in MEM low IgG FCS. Cell nuclei are shown in blue (DAPI), and HEV ORF2-positive foci are shown in green (α-ORF2 pAb rabbit serum and α-rabbit mAb AF488 2ndary; 10× objective in widefield microscopy).

Although existing HEV cell culture models are important achievements, these systems are limited in efficient viral spread and low viral titers (11). With the aim to improve production of HEV in tissue culture, we characterized extracellular and intracellular viral titers of the widely used Kernow-C1 p6 strain (gt 3) in different combinations of human hepatoma cell lines and media conditions for viral production and infection (Fig. 1*A*). HepG2 cells or a subclone thereof, HepG2/C3A cells, which were selected for strong contact inhibition of growth, were transfected with in vitro transcribed HEV p6_wt RNA. Each cell line was cultivated in either DMEM complete or a medium with lowered IgG levels (MEM low IgG FCS). After 7 d, extracellular and intracellular virus particles, resembling the enveloped and nonenveloped states of HEV, were harvested and used to inoculate both naïve cell lines cultivated in the 2 media. In total, we tested 32 different combinations (Fig. 1 *A* and *B*). Depending on the cultivation condition, infectious viral titers of intracellular-derived virus ranged from 5 × 10^2^ to 5 × 10^5^ FFU/mL and for extracellular virus from 2 × 10^1^ to 5 × 10^3^ FFU/mL as visualized by a heat map (Fig. 1*B*). Absolute viral titers with individual experimental data are shown in *SI Appendix*, Fig. S1*A*. A combination of transfection of HepG2 cells supplemented with DMEM complete and subsequent infection of HepG2/C3A in MEM low IgG FCS yielded the highest intracellular-derived viral titers (Fig. 1*B*). Immunofluorescence analysis of transfected cells revealed high percentages of HEV ORF2-positive cells at day 7 p.t. (*SI Appendix*, Fig. S1*B*). Of note, fluorescence intensity in transfected cells grown in MEM low IgG FCS was lower compared to DMEM, most probably due to decreased replication. In line with this finding, produced titers were notably lower in MEM low IgG FCS cultivated HepG2 and HepG2/C3A. Similarly, extracellular viral titers reached the optimum when virus production was performed in either HepG2 or HepG2/C3A cells supplemented with DMEM complete and infection was conducted in HepG2/C3A cells (Fig. 1*B*). Here we observed robust infection and replication of target cells indicated by ORF2 positivity (Fig. 1*C*).

The viral RNA determined in 50 ng of total RNA for the produced intracellular viral particles with the 4 different cell and media conditions (HepG2 cells with DMEM complete or MEM low IgG FCS and HepG2/C3A with DMEM complete or MEM low IgG FCS) was comparable ranging between 1 × 10^6^ and 3 × 10^6^ HEV copy numbers (*SI Appendix*, Fig. S1*C*). The results confirmed the beneficial effect of the low IgG FCS during the HEV infection process and not during viral production. For the extracellular-produced viral particles, RNA copy numbers were lower compared to the intracellular-derived particles resembling the infection data (*SI Appendix*, Fig. S1*C*).

A similar infection efficiency with the optimal conditions could be achieved in Huh7.5 cells (*SI Appendix*, Fig. S1*D*). These cells were not followed up upon, due to their RIG-I deficiency. Next, we applied these media conditions to the HEV cell culture model described by Schemmerer et al. (19), which is based on a persistently HEV gt3 47832c isolate-infected A549 cell line. Intracellular- and extracellular-derived viruses of these cells were harvested and used to infect either HepG2/3CA or A549/D3 cells, a A549 subclone selected for high permissiveness to HEV infection, in DMEM complete or MEM low IgG FCS media conditions. As depicted in *SI Appendix*, Fig. S1*E*, viral titers for intracellular-derived 47832c virus ranged between 5 × 10^4^ and 2 × 10^5^ FFU/mL in the different conditions and around 1 × 10^3^ FFU/mL for the extracellular-derived particles (*SI Appendix*, Fig. S1*E*). The low IgG FCS medium was superior over DMEM complete in the HepG2/3CA infection with intracellularly harvested particles, in line with the p6 isolate. However, for the infection of A549/D3 cells, the DMEM complete media resulted in higher viral titers. In conclusion, we established a simple HEV cell culture protocol based on the HEV Kernow-C1 p6_wt strain, which could also be confirmed with other HEV isolates and cell lines. The combination of the human hepatoma cell lines HepG2 supplemented with DMEM complete during virus production and HepG2/C3A in MEM low IgG FCS during virus infection allowed robust production of infectious HEVcc with titers over 10^5^ FFU/mL for the intracellular-derived particles (Fig. 1*A* and *SI Appendix*, Fig. S1*A*).

### *Trans*-Complementation of HEV RNA into Infectious HEVtcp.

**Fig. 2.**
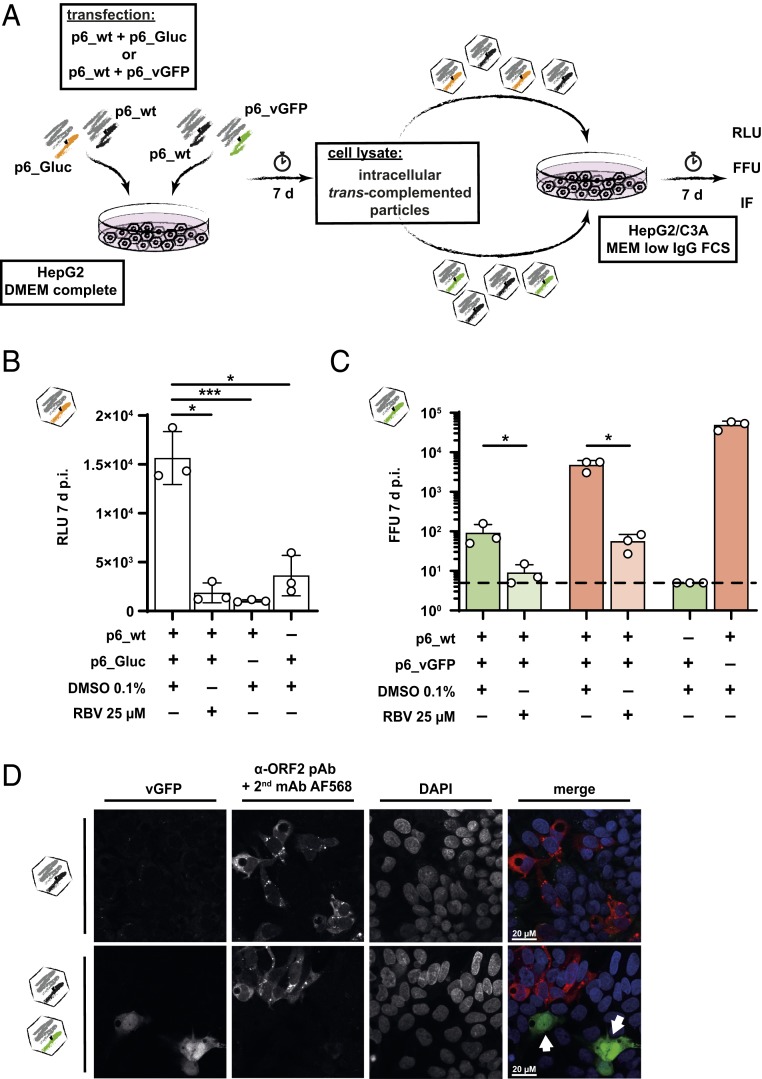
Production of infectious particles by *trans*-complementation of HEV reporter genomes. (*A*) Schematic representation of the workflow. HepG2 cells are transfected with equimolar amounts of either HEV p6_wt and p6_Gluc (*Gaussia* luciferase reporter) or p6_wt and p6_vGFP (venus green fluorescent protein) and incubated for 7 d. Assembled particles harboring either viral genome were purified from lysed cells and used to inoculate naïve HepG2/C3A. After an additional 7 d, infectivity was assessed via luminometer (relative light units [RLU]), immunofluorescence (IF), or ORF2 staining (FFU). (*B*) Infectivity of p6_Gluc *trans*-completed particles. Results of single experiments are shown as dots, and heights of bars indicate the mean of 3 independent biological replicates (RLU, linear *y* axis; *n* = 3 ± SD; *P < 0.05, ***P < 0.001, ANOVA followed by Dunnett’s corrected *t* test). RBV 25 µM was used to monitor effective replication in infected cells. (*C*) Infectivity of p6_vGFP *trans*-complemented virions was recorded either by microscopically counting vGFP-positive foci assessing infection events of *trans*-complemented particles only (green bars). Alternatively, ORF2 was stained with α-ORF2 pAb rabbit serum and a α-rabbit mAb AF568 (red bars) to assess p6_wt assembled viral particles. RBV 25 µM was used to monitor effective replication in infected cells (FFU, log *y* axis; *n* = 3 ± SD; *P < 0.05, ANOVA followed by Sidak’s corrected *t* test; dashed line, limit of quantification [LOQ]; titers below LOQ set to LOQ). (*D*) Representative fluorescence images of infection events following purification of assembled virions from lysed cells transfected with p6_wt only (*Upper*) or p6_wt and p6_vGFP cotransfected cells (*Lower*). VGFP-positive cells (first column) reflect infection of HepG2/C3A target cells with *trans*-complemented particles, while AF568-positive cells foci (second column) show p6_wt infection events in confocal microscopy. White arrows point to infection events with *trans*-complemented particles. (Scale bar, 20 µM.)

Based on the efficient production of HEVcc in vitro, we next aimed to develop a system that supports particle production by *trans*-packaging of subgenomic RNAs and therefore would allow the generation of viral-like particles with reporter activity. Furthermore, such an experimental system should be helpful to decipher mechanisms of HEV assembly and packaging, which is not well defined (20). To explore if assembly-deficient p6 genomes with deletions in the ORF2/3 can be rescued by *trans*-complementation, we cotransfected HEV reporter constructs with replaced ORF2 gene together with full-length HEV p6_wt genome as a helper virus RNA into HepG2 cells cultivated with DMEM complete. Seven days later, cell lysates containing intracellular infectious virus particles were harvested to infect naïve HepG2/C3A cells in optimized medium conditions. Successful *trans*-complementation of viral particles, termed HEVtcp, was assessed by determination of GFP or *Gaussia* luciferase (Gluc) activity (Fig. 2*A*). The assembly-defective HEV RNA encoding a *Gaussia* luciferase was encapsulated into infectious particles as evidenced by easily detectable reporter activity in the inoculated cells (Fig. 2*B*), which was controlled by transfection of p6_wt and reporter RNA alone. In addition, RBV treatment reduced transduction of reporter activity (Fig. 2*B*). Next, t*rans*-complementation of GFP reporter RNA demonstrated absolute HEVtcp titers of 1 × 10^2^ FFU/mL with simultaneous detection of the wild-type viral strain with 5 × 10^3^ FFU/mL of intracellular particles (Fig. 2*C*). Single transfections and RBV incubations served as controls.

The viral RNA detected with an ORF1-based PCR of the HEV *trans*-complemented cell lysates could be determined with 3 × 10^5^ copy numbers per 50 ng total RNA and was reduced by RBV treatment (*SI Appendix*, Fig. S2 *A* and *B*). The p6_wt viral loads, where no subgenomic reporter RNA was present, were higher compared to the cotransfection setting as expected. In the cell lysate of the p6_Gluc cotransfected conditions, RNA copy numbers of 2 × 10^4^ could be detected (*SI Appendix*, Fig. S2*A*). Similar results were observed for the GFP reporter replicon settings, with the exception of higher RNA copy numbers of the GFP replicon only cell lysates (*SI Appendix*, Fig. S2*B*).

To directly show that virus-like particles carrying the GFP reporter gene had productively infected the target cells, we assessed infection events at the single-cell levels using indirect immunofluorescence and flow-cytometry. Employing antibodies directed against ORF2, we detected cells expressing only ORF2 as well as cells expressing only GFP (Fig. 2*D* and *SI Appendix*, Fig. S2 *C* and *D*). These data suggest that besides viruses with full-length HEV genome, single round infectious particles containing the GFP reporter RNA had successfully infected the target cells (highlighted by white arrows) (Fig. 2*D*). The relative packaging efficiency of the GFP RNA in comparison to the p6_wt encoding RNA was analyzed via flow cytometry. As depicted in *SI Appendix*, Fig. S2 *C* and *D*, in the untreated HEVtcp sample a 1:60 ratio of the GFP reporter genome in comparison to the HEV p6_wt virus, indicated by the percentage of HEVcc- and HEVtcp-infected cells (*SI Appendix*, Fig. S2 *A* and *B*), was observed. Finally, we performed RNase A treatments of the intracellular- and extracellular-derived HEVtcp to demonstrate that the packaged genome is fully protected and no replicon-transfected cells were transferred. The RNase treatment did not result in a reduction of intracellular and extracellular HEVtcp titers, and only intracellular RNA that was not yet packaged into viral particles could be degraded (*SI Appendix*, Fig. S2 *E* and *F*). In summary, these results showed that HEVtcp particles are assembly- and secretion-competent and infectious. Taken together, a HEV *trans*-complementation system could be implemented enabling the analysis of HEV assembly and packaging.

### Impact of an RNA-Dependent RNA Polymerase Mutation on HEVcc Production.

**Fig. 3.**
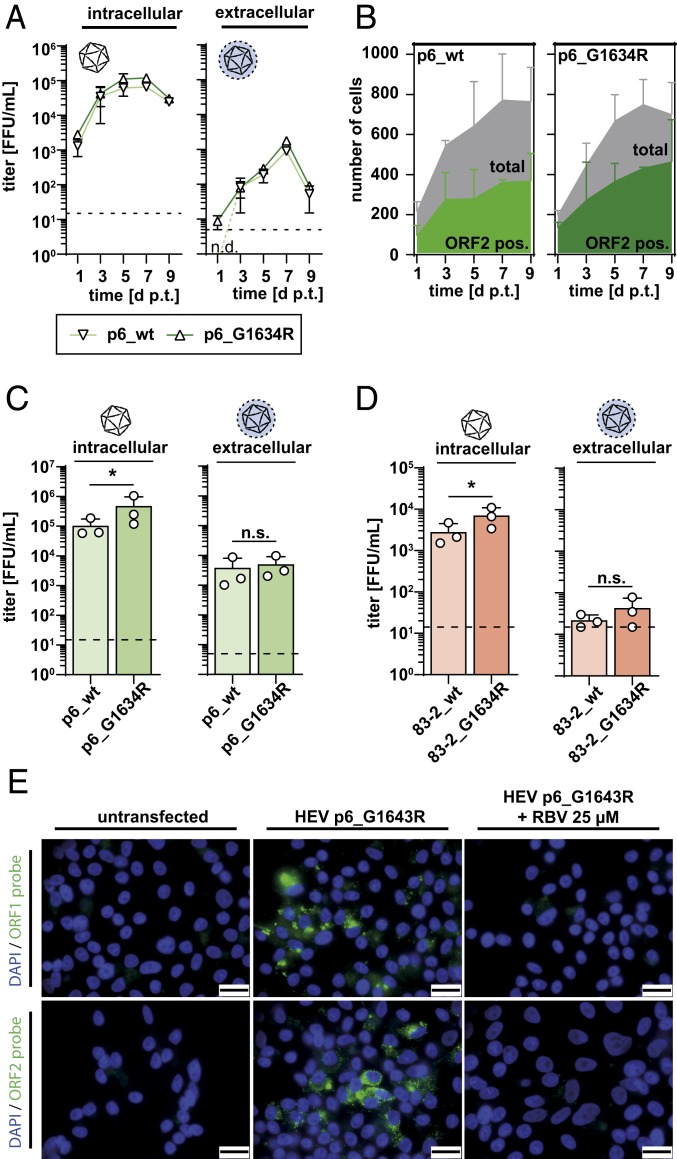
Introduction of the replication enhancing single nucleotide variant G1634R increases titers. (*A*) Kinetics of the production of intracellular (*Left*) and extracellular (*Right*) infectious particles assessed as FFU/mL (log *y* axes) over 9 d (linear *x* axes). Light green lines with inverted triangles represent the production in p6_wt HEV RNA transfected HepG2 cells, while dark green lines with triangles display the kinetics in the p6_G1634R mutant transfected cells (dashed line, LOQ; *n* = 2 ± SD; n.d., not detected). (*B*) Number of HEV ORF2-positive cells (green areas) in p6_wt (*Left*) or p6_G1634 mutant (*Right*) HEV RNA transfected cultures monitored over time (1 representative IF picture evaluated per construct and day; mean of 2 independent biological replicates; *n* = 2 ± SD). (*C*) Titer of infectious particles represented as FFU/mL (log *y* axes) harvested from lysed cells (*Left*) or supernatant (*Right*) of p6_wt (light green bars) or p6_G1634R (dark green bars) RNA transfected cells. Titers of single experiments are presented as white dots, and bars display the mean of 3 independent biological experiments (dashed line, LOQ; *n* = 3 ± SD, *P < 0.05 in a ratio paired *t* test, n.s., not significant). (*D*) Titer of infectious particles represented as FFU/mL (log *y* axes) harvested from lysed cells (*Left*) or supernatant (*Right*) of 83-2_wt (light red bars) or 83-2_G1634R (dark red bars) RNA transfected cells. Titers of single experiments are presented as white dots, and bars display the mean of 3 independent biological experiments (dashed line, LOQ; titers below LOQ set to LOQ; *n* = 3 ± SD, *P < 0.05 in a ratio paired *t* test, n.s., not significant). (*E*) Fluorescence pictures of RNA in situ hybridization of HEV p6_G1634R ORF2-positive strand subgenomic RNA. HepG2 cells were either mock-transfected (*Left*) or transfected with HEV p6_G1634R virus and left untreated (*Middle*) or treated with RBV (*Right*). (Scale bars, 20 µm.)

Ribavirin treatment failures were linked to the selection of a distinct HEV polymerase variant (G1634R) in some chronically HEV-infected patients resulting in increased replication fitness (13, 21, 22). To assess if this mutation further increases viral production in tissue culture, we used the described protocol and determined virus production of p6_wt and p6_G1634R in a time-dependent manner (Fig. 3*A*). For both strains, newly produced infectious viral particles reached a maximum 7 d after transfection with a reproducible slight increase for the mutant viral strain (Fig. 3*A*). Immunofluorescence staining of ORF2 indicated viral spread in the HepG2 cells with a high rate of infected cells and an increase of ORF2-positive cells over time (*SI Appendix*, Fig. S3*A*). HEV RNA determination for the intracellular-derived particles revealed higher levels for the mutant viral strain, which increased over time (*SI Appendix*, Fig. S3*E*). For the extracellular-derived particles, copy numbers plateaued at 10^6^ per 50 ng total RNA (*SI Appendix*, Fig. S3*E*). Furthermore, mean fluorescence intensities of ORF2 staining were obtained from a 5-pixel-wide cytoplasm ring (cytoring) following segmentation of DAPI stained nuclei using CellProfiler (23). To distinguish noninfected cells from infected cells a minimum intensity threshold was determined (*SI Appendix*, Fig. S3*B*), which showed a higher number of antigen-positive cells in the case of p6_G1634R, which accumulated over time (Fig. 3*B*). These results were supported by an analysis of the ratio of ORF2-positive cells to the total cell number (*SI Appendix*, Fig. S3*C*). Indeed, we also observed a trend to bigger foci sizes at later time points in p6_G1634R-transfected cells, assessed by automatically counting the number of nuclei per focus (*SI Appendix*, Fig. S3*D*). Quantification of the viral titers 7 d p.t. demonstrated significantly higher titers of intracellular infectious particles of 4.7 × 10^5^ FFU/mL for the p6_G1634R compared to around 1 × 10^5^ FFU/mL for the p6_wt titers, with 1 viral stock of even 1 × 10^6^ FFU/mL (Fig. 3*C*). In the case of the extracellular-derived particles, only a slight increase from 3.8 × 10^3^ FFU/mL for the wild-type strain to 4.9 × 10^3^ FFU/mL was observed for the strain with the mutation in the RNA-dependent RNA-polymerase (Fig. 3*C*). These results were confirmed by an analysis of the HEV RNA copy numbers (*SI Appendix*, Fig. S4*A*). To universalize our results, we analyzed another gt 3 strain (HEV83-2‐27) that lacks the insertion in the hypervariable region (HVR) (24). Introduction of the mutation in a *Gaussia* reporter replicon revealed no enhancement of viral fitness as previously demonstrated for the p6 strain (13, 22), neither when assessing HEV RNA copy numbers (*SI Appendix*, Fig. S4*B*) nor when measuring luciferase activity (*SI Appendix*, Fig. S4*C*). However, using the full-length system, intracellular and extracellular viral titers of 2.8 × 10^3^ and 2.2 × 10^1^ FFU/mL could be produced. Introducing the G1634R mutation significantly improved particle production to 7 × 10^3^ FFU/mL for intracellular virions (Fig. 3*D*), while again for extracellular particles only a duplication of infectious units was noted. The specific infectivities, defined as number of RNA copies per infection event, were comparable between HEV p6_wt and p6_G1634R with intracellular-derived virus ranging from 4.0 to 4.8 × 10^−2^ and for extracellular virus from 0.9 to 2.4 × 10^−2^ FFU/RNA copy, respectively. In the case of the 83-2-27 strain the specific infectivities were lower, ranging from 3.0 to 3.7 × 10^−3^ (intracellular) and 2.9–5.1 × 10^−5^ FFU/RNA copy (extracellular).

These data indicate that the G1634R mutation in the RNA-polymerase of p6 could further significantly improve viral spread representing the strain with the highest efficiency in virus production. Viral infectivity could be propagated over several cell passages but, however, was reduced by 1 order of magnitude after 5 passages (*SI Appendix*, Fig. S4*D*). The produced intracellular-derived viral particles of p6_G1634R could be neutralized at high dilutions of a WHO standard harboring HEV-specific antibodies, while the membrane-wrapped extracellular particles required higher concentrations for inhibition (*SI Appendix*, Fig. S4*E*). To visualize RNA replication of p6_G1634R in transfected HepG2 cells, specific probes against the positive strand RNA of ORF1 and ORF2 were designed and detected in a fluorescence in situ hybridization (FISH) assay. As depicted in Fig. 3*E*, both ORF1- and ORF2-encoding RNA could be detected in p6_G1634R replicating cells, which was ablated in RBV-treated cells (Fig. 3*E*). This method can be employed to study colocalization of the distinct subgenomic RNAs and is of special interest in the view of the fact that potent ORF1-specific antibodies for immunofluorescence are lacking.

Next, we examined the density of intracellular- and extracellular-produced p6_wt and p6_G1634R particles by iodixanol gradient centrifugation. Gradient fractions were collected after centrifugation and analyzed for the presence of HEV RNA and infectious virions. As depicted in *SI Appendix*, Fig. S5*A*, p6_wt and mutant intracellular-derived particles peaked at high densities of around 1.25 g/mL indicative of mainly nonenveloped viruses, while for extracellular-derived particles, RNA levels at lower densities were observed (*SI Appendix*, Fig. S5). For the extracellular particles, infectious virus could be detected between densities of 1.05 and 1.1 g/mL with no major differences between the wild-type and the mutant strain. Collectively, these results indicate that high-titer p6_wt and p6_G1634R display similar biophysical properties.

### Infection of Humanized Mice with HEVcc.

**Fig. 4.**
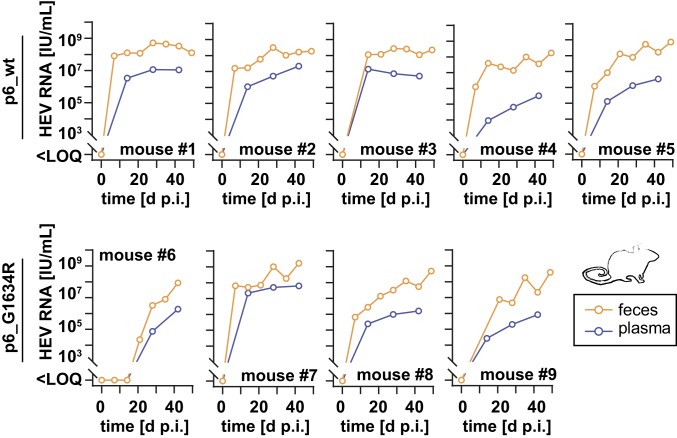
HEVcc establish high-titer infections in humanized liver chimeric mice. Humanized mice were inoculated intraperitoneally with either intracellular cell culture-derived HEV p6_wt (*Upper*; 5 mice) or p6_G1634R (*Lower*; 4 mice). HEV RNA (log *y* axes) was periodically measured in plasma (purple lines) and feces (orange lines). LOQ, limit of quantification.

Recently, several studies demonstrated that human liver chimeric mice can be infected with HEV and are useful tools for studying chronic HEV infection (25–28). However, previous inoculations of mice with HEVcc particles resulted in low viral titers in stool specimens and were negative in plasma samples (27). To investigate the infectivity of the optimized cell culture system-derived HEVcc, humanized mice were intraperitoneally infected with 2.5 × 10^4^ FFU/mouse of intracellular-derived p6_wt or p6_G1634R virus. As depicted in Fig. 4, HEV RNA of both strains were detectable in high copy numbers in the feces of infected animals over several weeks without decline (Fig. 4). Importantly, this was also the case for plasma samples with levels up 10^8^ RNA IU/mL. The p6_G1634R resulted in similar infection levels compared to the wild-type strain, with the exception of mouse 6, which showed a delayed viral growth (Fig. 4). In summary, our HEVcc cell culture system allowed efficient infection of humanized mice, which enabled detection of viral RNA in murine plasma samples in addition to feces.

### Infection of Primary Human and Porcine Hepatocytes with High-Titer HEVcc.

**Fig. 5.**
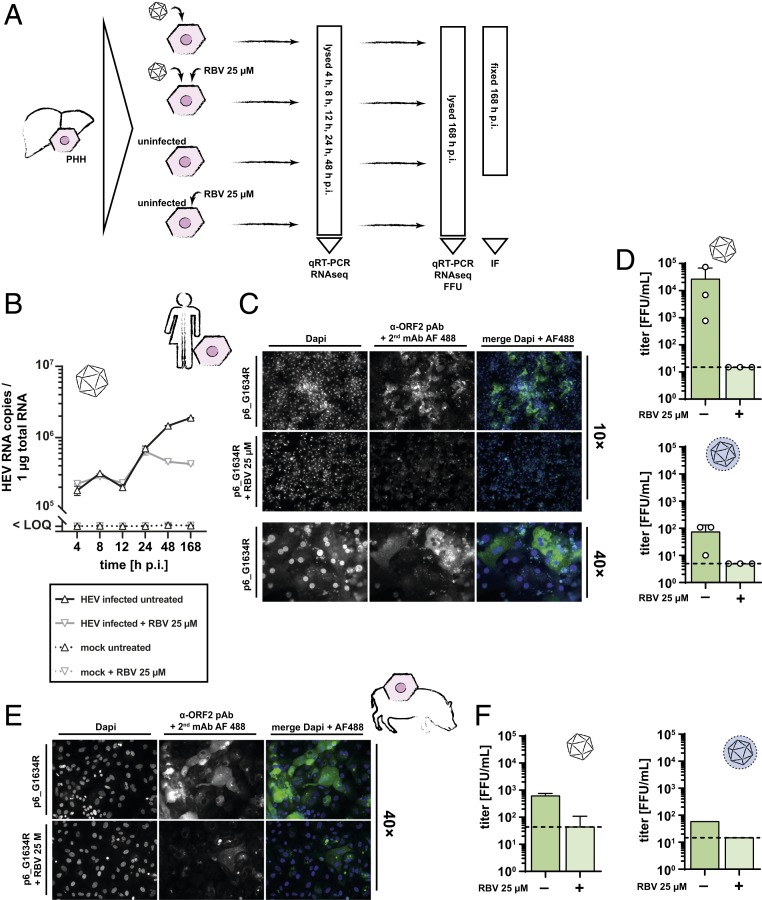
HEVcc establish productive infections in primary human and porcine hepatocytes. (*A*) Schematic representation of the PHH experiment. PHH were plated on 6-well plates and inoculated with intracellular-derived HEV p6_G1634R (MOI = 1) with or without the administration of RBV 25 µM. After 4, 8, 12, 24, 48, and 186 h p.i., cell lysates were harvested and analyzed via qRT-PCR and RNAseq. The PHH were additionally used for IF staining and FFU count 168 h p.i. (*B*) Replication of HEV RNA in PHH was monitored via qPCR (log *y* axis) for 7 d (categorical *x* axis). Black solid line represents the course of HEV RNA in infected, untreated cells, while the gray solid line depicts the course in infected but RBV 25 µM-treated cells. Dotted lines exemplify mock-infected cells. Triangles and inverted triangles mark the mean of 2 technical replications (*n* = 2 ± SD). (*C*) Representative fluorescence images of HEVcc-infected PHH stained with α-ORF2 pAb rabbit serum and α-rabbit mAb AF488 2ndary (widefield microscopy). Administration of RBV served as control. (*D*) Newly produced viral particles (intracellular, *Upper*; extracellular, *Lower*) were recovered from productively HEVcc-infected, lysed PHH and used to inoculate naïve HepG2/C3A target cells. Assembly and infectivity were assessed by counting ORF2-positive foci (titer; log *y* axes). Titers of single experiments are presented as white dots, and green bars display the mean of 3 independent titrations (dashed line, LOQ; titers below LOQ set to LOQ; *n* = 3 ± SD). (*E*) Representative fluorescence images of HEVcc-infected PPH stained with α-ORF2 pAb rabbit serum and α-rabbit mAb AF488 2ndary (widefield microscopy). Administration of RBV served as control. (*F*) Newly produced viral particles (intracellular, *Left*; extracellular, *Right*) were recovered from productively HEVcc-infected, lysed PPH and used to inoculate naïve HepG2/C3A target cells. Assembly and infectivity were assessed by counting ORF2-positive foci (titer; log *y* axes). Titers are presented as green bars (dashed line, LOQ; titers below LOQ set to LOQ; error bars indicate SD of titration assay).

Adult PHH are the target of HEV in vivo and represent the most authentic cell culture system for hepatotropic viruses. To investigate the potential of the high-titer HEVcc particles to infect PHH, we challenged these cells with intracellular-derived p6_G1634R particles with a MOI of 1. After 4, 8, and 12 h, resembling early to intermediate cellular responses to viral challenge, as well as after intermediate to late responses (24, 48, and 168 h postinfection [p.i.]), we measured viral replication and propagation, along with temporal transcriptional changes in the infected primary cells (Fig. 5*A*). Kinetics of viral replication determined by RT-PCR showed increasing RNA levels 24 h after infection, which peaked at the end of the experiment 168 h p.i. (Fig. 5*B*). Administration of 25 µM RBV inhibited HEV replication. Immunofluorescence analysis of infected cells demonstrated a high rate of about 30% ORF2-positive PHH, determined with CellProfiler as described above (Fig. 5*C*). Replication of ORF1 was again visualized via FISH (*SI Appendix*, Fig. S6). Immunofluorescence staining of ORF2 protein and in situ hybridization of ORF1 RNA proved efficient viral transcription and translation in PHH. Additionally, we could harvest newly produced infectious virions from infected PHH to inoculate naïve HepG2/C3A. Using this approach, we measured mean viral titers of 2.7 × 10^4^ FFU/mL for intracellular-derived HEV, for extracellular-derived HEVcc viral infectivity was 100-fold lower. Propagation of both was abrogated by RBV treatment (Fig. 5*D*). As pigs are the main natural reservoir for HEV gt 3, we next inoculated primary porcine hepatocytes (PPH). As depicted in Fig. 5*E*, HEVcc infection of PPH resulted in robust infection visualized by immunofluorescence microcopy and was ablated by RBV treatment. Presence of infectious particles could be demonstrated for both types of viruses; however, virus yields were lower compared to the PHH (Fig. 5*F*). Taken together, HEVcc particles produced in an optimized cell culture system were able to efficiently infect PHH as well as PPH.

### Transcriptional Network Engaged upon HEV Infection of Primary Hepatocytes.

**Fig. 6.**
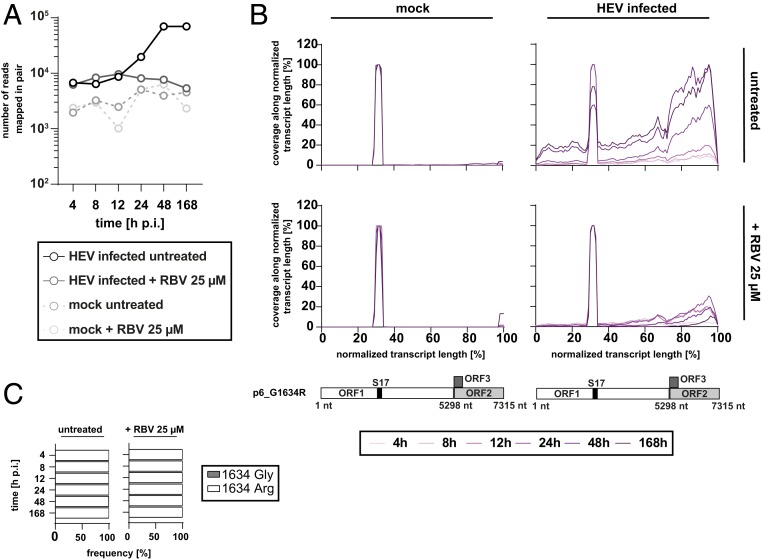
Total RNAseq of HEVcc-infected PHH reveals a replication-specific increase of distinct HEV genome transcripts. (*A*) Total RNA extracted from HEVcc p6_G1634R-infected PHH (compare Fig. 5) of one representative donor were supplied to Illumina RNAseq, and HEV RNA abundance (log *y* axis) was monitored over time (categorical *x* axis). Black solid line and dots represent the course of HEV RNA in infected, untreated cells, while the gray solid line and dots depict the course in infected but RBV 25 µM treated cells. Dashed lines and dots exemplify mock-infected cells. (*B*) Normalized coverage of mapped reads (linear *y* axes) along the HEV genome (linear *x* axes) in HEVcc-infected (*Right*) and mock-infected PHH (*Left*). Hepatocytes were either treated with RBV 25 µM (*Lower*) or left untreated (*Upper* ). Increments of purple lines indicate the change in coverage over the monitored time. Below the plots, a schematic of the HEV p6 genome acknowledges the positioning of the 3 ORF and the S17 insertion. (*C*) Genomic stability of the introduced G1634R variant over the time of the experiment (categorical *y* axis) depicted as frequency of amino acid residues (linear *x* axes) under RBV 25 µM treatment and untreated conditions. White bars represent the arginine variant, and gray bars depict frequency of the glycine variant.

The established HEV infection model in PHH was further applied to study host responses upon infection via transcriptomic analysis. PHH were infected with p6_G1634R at a MOI of 1 and monitored over time for 168 h (Fig. 5*A*). Total RNA was extracted and supplied to Illumina RNAseq. First, reads that mapped to the viral genome were analyzed. As observed in the previous experiment, HEV RNA accumulated over the course of infection, reflected by the increase in number of RNAseq reads that map to the viral reference genome, and were reduced by RBV treatment (Fig. 6*A*). High background levels also identified in the mock-infected control PHH arise from reads derived from the host’s ribosomal subunit S17 erroneously mapping to the HEV p6 reference genome. Same holds true for the coverage plots, where peaks of coverage in all 4 setups were identified resembling mismatches at the locus of the S17 insertion on the HVR of p6 (29) (Fig. 6*B*). For HEV-infected samples, viral transcripts increased over time and peaked at 48 h p.i. (Fig. 6*B*). Interestingly, HEV transcripts encoding ORF2/3 were more abundant than ORF1 genomic RNA (Fig. 6*B*). The coverage of mapped reads was reduced in the RBV control (Fig. 6*B*). Analysis of the genomic stability of the introduced G1634R variant demonstrated no change over the course of infection in PHH (Fig. 6*C*).

**Fig. 7.**
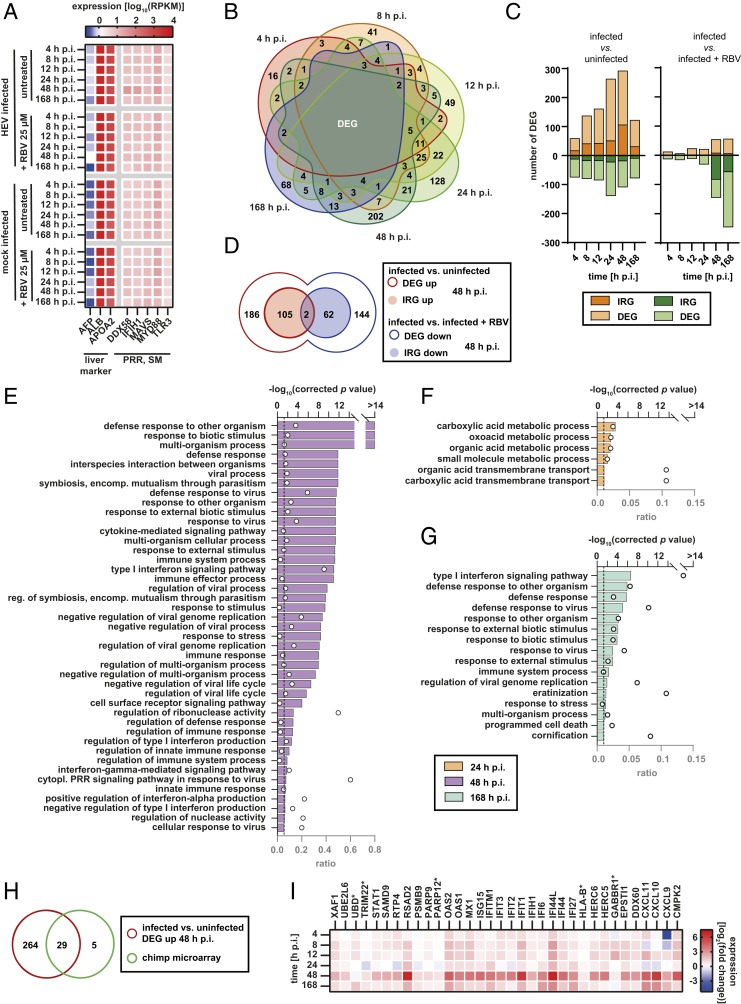
Transcriptional responses in PHH to HEVcc infection. (*A*) Heat map of normalized transcript expression (reads per kilobase per million base pairs mapped [RPKM]) of biomarker of adult hepatocytes and PRRs as well as SM in HEVcc-infected or mock-infected cells treated with RBV 25 µM or left untreated over time. (*B*) Venn diagram of significant DEGs at each monitored time point in HEVcc-infected PHH compared to uninfected PHH. (*C*) Total number of significant DEG (linear *y* axes) up- (light orange bars) or down-regulated (light green bars) over time (categorical *x* axes) in HEVcc-infected PHH compared to either uninfected hepatocytes (*Left*) or infected but RBV 25 µM treated cells (*Right*). Fractions of previously described IRGs are colored darker. (*D*) Venn diagram of significant up-regulated DEG (red line, white fill) and IRG (red line, red fill) in HEVcc-infected PHH compared to uninfected PHH and significant down-regulated DEG (blue line, white fill) and IRG (blue line, blue fill) in HEVcc-infected PHH compared to infected but RBV-treated PHH. (*E*–*G*) Representation of analysis of significant enriched (Bonferroni corrected *P* value < 0.05, dashed line, upper linear *y* axis) pathways in HEVcc-infected PHH compared to uninfected PHH. Pathways are ordered according to significance with color of bars representing time point of enrichment: (*E*) pink, 48 h p.i., (*F*) orange, 24 h p.i., and (*G*) cyan, 168 h p.i. Open circles depict the number of regulated genes as ratio of the total number of genes assigned to the respective pathway (lower linear *x* axis). (*H* and *I*) Comparison of DEG to genes previously identified in HEV-infected chimpanzees (31). (*H*) Venn diagram displaying 29 DEG overlapping in both studies; 5 genes differentially regulated in HEV-infected chimpanzees were not significantly regulated in HEV-infected PHH (marked with asterisks). (*I*) All 34 DEG were plotted that were regulated on average at least 1.2-fold in the mentioned study in alphabetical order. Color code represents the fold change (FC) of the expression in HEVcc-infected PHH compared to uninfected cells (log_2_FC) at different time points.

Next, we investigated the transcriptional host response upon HEV infection in PHH. High expression of hepatocyte markers ALB and APOA2 and minimal expression of the fetal hepatocyte marker AFP confirmed the maintenance of mature hepatocyte phenotype in plated PHH during the course of the experiment (Fig. 7*A*). Furthermore, intrinsic expression of pattern recognition receptors (PRRs) DDX58 (also known as RIG-I), TLR3, and IFIH1 (also known as MDA5) were detected, in addition to downstream signaling molecules (SM) MYD88 and MAVS. These data support the physiological ability of the PHH to detect viral infections and induce innate immune signaling cascades (Fig. 7*A*). Analysis of significant differentially expressed genes (DEGs) in infected PHH compared to uninfected cells revealed distinct expression patterns for each time point with low overlap (Fig. 7*B*). The highest number of DEGs was observed at 48 h p.i., which is in line with the peak level of viral replication (Fig. 6 *A* and *B*). Next, we compared the identified DEGs with previously described IFN-regulated genes (IRGs) (30). All IRGs significantly up- (*SI Appendix*, Fig. S7*A*) or down-regulated (*SI Appendix*, Fig. S7*B*) at least at one time point during the experiment are summarized in *SI Appendix*, Fig. S7 *A* and *B*. The highest up-regulation of IRGs was observed after 48 h pointing to a strong antiviral state in PHH upon HEV infection (Fig. 7*C*). Inhibiting HEV replication by RBV resulted in only a mild up-regulation of IRGs and DEGs. Importantly, a strong down-regulation of IRGs and DEGs was noted, which was induced by the antiviral therapy (Fig. 7*C*). In line, this down-regulation was also observed in uninfected but RBV-treated cells (*SI Appendix*, Fig. S7*C*). When comparing transcript expression at different time points of the experiment with the expression at 4 h, we noted a steady increase in the number of DEGs. This was true for the infected (*SI Appendix*, Fig. S7*D*) as well as for the uninfected PHH (*SI Appendix*, Fig. S8*C*) resembling the differentiation of primary cells once plated. A comparison of DEGs and IRGs at 48 h p.i. up-regulated in the infected PHH and down-regulated in the infected and RBV-treated cells revealed a regulation of distinct genes that did not overlap (Fig. 7*D*). Gene ontology (GO) enrichment analyses of biological processes of all significant DEGs of HEV-infected PHH compared to uninfected cells over the course of infection identified pathways involved in the cells defense responses at 24, 48, and 168 h p.i. (Fig. 7 *E*–*G*). Same observations in the comparison between the infected PHH and infected but RBV-treated cells (*SI Appendix*, Fig. S8 *A* and *B*) point to a specific regulation of genes caused by the viral infection and replication. Furthermore, the type I IFN signaling pathway (GO:0060337) and cytoplasmic PRR signaling pathway in response to virus (GO:0039528) are among the pathways with the highest ratio of significantly differentially regulated genes to the total number of genes associated to the pathways, indicating the competence of PHH to sense viral infections and trigger IFN release and innate immune response (Fig. 7*E*). Interestingly, at 24 h p.i., mainly genes associated with metabolic processes were significantly regulated (Fig. 7*E*). The fold changes in expression of members of the pathway defense response (GO:0006952) as representative of the primary regulated pathways are depicted in *SI Appendix*, Fig. S6*B*. Ribavirin treatment in uninfected PHH interfered with the expression of certain genes (*SI Appendix*, Fig. S7*C*) of predominantly metabolic pathways not involved in innate immune responses (*SI Appendix*, Fig. S7*E*). The PHH transcriptome changed also over time in the mock-infected cells with mainly genes belonging to cellular processes and metabolic processes and minor IRG-related genes (*SI Appendix*, Fig. S8*C*). Using microarray analysis, Yu et al. (31) previously studied the host response to HEV infection in multiple experimentally infected chimpanzees. They identified several significantly up-regulated genes that, with the exception of 5 genes, were also up-regulated in inoculated PHH, predominantly 48 h p.i. (Fig. 7*F*).

In summary, these results demonstrate the ability of our HEVcc virions to establish robust infections and replicate in plated adult PHH. Furthermore, we show that PHH are able to sense HEV infections and trigger a temporally structured transcriptional defense response.

## Discussion

Although many groups have successfully propagated various HEV strains in different cell lines, viral replication often remained low and could often only be detected by sensitive PCR methods. Another limitation relates to the distinct phenotypic characteristics of the cell lines utilized, including nonhepatic lineages (e.g., A549), recombinant manipulations (e.g., PLC/PRF/5 expressing hepatitis B viral genes), or the lack of a proper immune response (e.g., Huh-7.5). The historical lack of an efficient in vitro culture system severely restricts HEV research. As a consequence, off-label RBV and pegIFN-α remain the treatment of choice in chronic infections with currently no drugs being approved that specifically target HEV (7).

Here we report the establishment of a simple yet robust cell culture HEV infection system. Our model is based on the HEV genotype 3 Kernow-C1 p6 strain and the human hepatoma cell lines HepG2 and HepG2/C3A combined with different media conditions. The HEV p6 strain was originally isolated by Shukla and colleagues using fecal samples obtained from an HIV patient chronically coinfected with HEV (32). The virus was semipurified from the feces and used to inoculate several cell lines. After 6 passages, an adapted virus was isolated and termed Kernow-C1/p6. Sequence analysis data showed an insertion of 58 amino acids of the human ribosomal subunit S17 in the HVR of ORF1 (29). A minority of viral genomes containing the S17 insertion was identified in fecal samples from which the original Kernow-C1 strain was isolated, indicating that the insertion was obtained in the infected host and was not a cell culture artifact (29). By testing 32 different cell and media conditions, we identified the best combination for the production of highly infectious HEVcc, i.e., when HepG2 cells supplemented with DMEM were transfected and HepG2/C3A cells cultured with MEM low IgG FCS were infected with the harvested intracellular particles (Fig. 1). HepG2/C3A cells were selected for strong contact inhibition of growth, which seemed to favor the HEV propagation after infection. The low IgG FCS was already used in HEV clearance studies by Farcet et al. (33) and probably shows less interference in the HEV entry pathway compared to other FCS charges. Comparably, in another published HEV cell culture system the authors inoculate target cells completely without the addition of FCS (34). However, further investigations are required to understand the effect of FCS products during HEV infection. In line, a recent study with the aim to isolate virus from human clinical specimens also described distinct medium supplements and the combinations thereof to increase viral loads in cell culture (19). Of note, by harvesting virions from the supernatant as well as from lysed cells, we were able to handle both enveloped and nonenveloped virus. In the future, this offers the opportunity not only to study fecal–oral transmission events caused by nonenveloped virions. In clinical transfusion and transplantation settings, the transmission of quasi-enveloped particles might be of more relevance as this state is the proposed state for HEV circulating in the blood stream.

The successful production of HEVtcp as single round infectious particles may further prove valuable for vaccination approaches in the future (Fig. 2). Since deletion of large parts of the ORF2/3 coding region was possible, this portion of the viral genome does not contain crucial *cis*-active elements required for packaging. These results confirmed and extended previous findings of Ding et al., who established a HEV *trans*-complementation approach based on stable packaging cells to identify a viroporin function of ORF3 (35).

The introduction of a mutation in the RNA-dependent RNA polymerase (G1634R) allowed an even further increase in viral titers of intracellular-derived particles, which was confirmed in another gt 3 strain (Fig. 3). Generally, the optimized protocol was adaptable to this HEV83-2 strain, although viral titers did not reach p6 levels. This is most probably due to less efficient replication of the strain compared to p6 (16). The G1634R mutation was originally identified as single nucleotide variant in chronically HEV-infected transplant patients undergoing treatment failure. Subsequent analysis revealed increased replication rates in vitro, while the RBV sensitivity was unmodified (13, 22). Similar specific infectivities between the p6_wt and p6_G1634R underlined this replication-dependent phenotype (Fig. 3). Characterization of the biophysical properties of high-titer produced p6_wt and p6_G1634R demonstrated comparable features in RNA copy numbers (*SI Appendix*, Fig. S5). In contrast to previous studies (26–28), these viral particles were able to replicate in humanized mice to high RNA levels detectable in plasma (Fig. 4) creating the opportunity to study a genetically defined virus in an HEV pathogenesis and vaccine model.

Cancer cell lines can be advantageous for culturing HEV due to easy handling, robustness, and availability, although tumor-derived cell lines may not faithfully recapitulate some cellular pathways compared to primary cells. Thus, stem cell-derived cellular systems or primary cells offer a more authentic system for studying HEV (36). Inoculation of PHH and importantly PPH cultures with high titer HEVcc allowed a robust infection in these cells determined by RT-PCR and immunofluorescence assays as well as a high de novo virus production (Fig. 5). These results represent data of robust HEV infection of PPH, creating the opportunity to study species-specific aspects of the viral life cycle in primary cells. So far, only a porcine embryonic stem cell-derived cell line has been developed for HEV in vitro studies (37).

To elucidate the applicability of the primary cells to study virus–host interactions, we applied RNAseq to infected PHH. First, we mapped the reads to the viral genome (Fig. 6). Interestingly, the ORF2/3-specific transcripts were far more abundant than the ORF1-encoding transcript suggesting a quantitative regulation of HEV protein expression during viral replication. This finding might explain the difficulty to detect ORF1 expression in HEV-positive samples (38). As the molecular mechanisms associated with HEV replication and cellular antiviral responses against HEV are only rudimentary understood, we determined the transcriptional landscape induced by HEV infection in PHH via RNAseq (Fig. 7). HEV-induced regulation of genes was timely structured in adult PHH with minimal overlap observed between the time points. Of note, PHH can have a donor to donor difference in the transcriptional response, which can influence the individual transcriptional landscape. Therefore, we compared our results to a microarray analysis by Yu et al., who previously studied the host response to HEV in multiple experimentally infected chimpanzees (31). Interestingly, we observed a high overlap between the genes significantly up-regulated in PHH 48 h p.i. and up-regulated in the liver of the infected chimpanzees (Fig. 7*F*). By comparing mock-treated and RBV-treated PHH, both HEV inoculated or not, we show that RBV leads to a repression of specific genes. In the case of HCV, similar observations have been made, where RBV down-regulates abnormally preactivated ISGs following HCV infection of PHH, which then restored IFN-responsiveness in the hepatic environment (39).

In conclusion, this improved HEVcc system provides a powerful tool for understanding basic HEV infection biology in various human hepatocyte systems and should accelerate the discovery of antiviral drugs and vaccines.

## Supplementary Material

Supplementary File
