# Supplementary material for: Robust hepatitis E virus infection and transcriptional response in human hepatocytes
Source: Proc Natl Acad Sci U S A. 2020 Jan 2;117(3):1731–41. doi: 10.1073/pnas.1912307117 (PMC6983376; doi:10.1073/pnas.1912307117)
Supplement: Supplementary File [file pnas.1912307117.sapp.pdf]

Supporting information for

Robust hepatitis E virus infection and transcriptional response in human hepatocytes

Daniel Todt[1,2,3*#], Martina Friesland[2*], Nora Moeller[1,2], Dimas Praditya[1,2], Volker Kinast[1], Yannick Brüggemann[1], Leonard Knegendorf[1], Thomas Burkard[1] Joerg Steinmann[4,5], Rani Burm[6], Lieven Verhoye[6], Avista Wahid[2], Toni Luise Meister[1], Michael Engelmann[1], Vanessa M. Pfankuche[7], Christina Puff[7], Florian Vondran[8], Wolfgang Baumgärtner[7], Philip Meuleman[6], Patrick Behrendt[2,9,10], Eike Steinmann[1,2#]

- Materials and Methods
- Supplementary Figures
- References

Materials and Methods

Compounds and reagents

Ribavirin (RBV) was received from Sigma Aldrich, St. Louis, MO, USA. For neutralization the WHO reference reagent for HEV antibody (NIBSC code: 95/584) was used in dilutions indicated. All compounds were dissolved, stored and diluted according to manufacturer's recommendations.

Cell culture

The human liver cell lines HepG2 and Huh-7.5 were cultured in Dulbecco's modified Eagle's medium (DMEM) (Gibco, Thermo Fisher Scientific, Schwerte, Germany) supplemented with 10% FCS (GE Healthcare, Munich, Germany (Ref A15-151, Lot A15111-2028) or Capricorn Scientific, Ebsdorfergrund, Germany (Ref FBS11-A, Lot CP16-1377) or Gibco (Ref 10270-106, Lot 41Q1820K)), 100µg/mL of streptomycin and 100 IU/mL of penicillin (Gibco), 2 mM L-glutamine and 1% nonessential amino acids (Gibco) (DMEM complete). The HepG2/C3A subclone was cultured in Eagle´s minimum essential medium (MEM with glutamine, Gibco), 10% ultra-low IgG FCS (Gibco, Ref 16250-078, Lot 1939770), 2 mM L-glutamine, 100 µg/mL gentamicin, 1 mM sodium pyruvate and 1% nonessential amino acids (Gibco) (MEM low IgG FCS medium).

MEM Eagle medium was also used for the culture of A549 cells which are persistently infected with the 47832c strain and the A549/D3 cells, a subclone of the A549 cell line (1). For the persistently infected cells, MEM was complemented with 2% FCS, 100 µg/ml gentamicin (Gibco), 2 mM L-glutamine and 1% non-essential amino acids, for the A549/D3 with 10% FCS, 50 µg/ml gentamicin, 2 mM L-glutamine and 1% non-essential amino acids. HepG2 and HepG2/C3A cells were further grown on rat collagen-coated (SERVA Electrophoresis GmbH, Heidelberg, Germany) culture plates. Cells were kept at 37°C in a 5% (vol/vol) CO2 incubator.

Primary human hepatocytes (PHH) were isolated from liver specimens, plated at a density of 1.3×10^6 on collagen-coated 6-well dishes, and kept in hepatocyte culture medium (William´s medium E) as described (2). Porcine hepatocyte isolation and culture was performed using the perfusion technique as previously applied in humans (2). Briefly, fresh liver specimen were flushed once with 500 mL washing buffer containing 2.5 mM EGTA, followed by recirculating perfusion with digestion buffer containing 0.05% collagenase (Roche P). The tissue then was mechanically disrupted, the resulting cell suspension poured through a gauze-lined funnel and

centrifuged with subsequent washing of the cell pellet using ice-cold PBS (50×g, 5 min., 4°C). Cells were then re-suspended in William's medium E (Biochrom AG) supplemented with 1 μM insulin, 1 μM dexamethason/fortecortin, 100 U/mLpenicillin, 100 μg/mLstreptomycin, 1 mM sodium pyruvate, 15 mM HEPES buffer, 4 mM L-glutamine, 5% FCS and 0.05 mmol/l β-mercaptoethanol.

Quantity and viability of cells were determined by trypanblue exclusion test. Hepatocytes were cultured using collagen-precoated 24-well plates, seeding at a concentration of 3×10^5viable cells per well. Sixteen to eighteen hours after plating, culture medium was changed to remove dead and non-adherent cells.

HEV 47832 strain infectious virus production assay

A549 cells persistently infected with the HEV gt 3 47832c strain were used to produce infectious HEV as described (1). Confluent cells were passaged 1:2 in DMEM complete and seven days later the supernatant containing the extracellular virus was centrifuged to remove cells. Intracellular virus was harvested as performed in the HEV infectious virus production assays and also resuspended in a five times lower volume of medium compared to the harvested supernatant. Viral titers were determined by serial dilution in either DMEM complete or MEM low IgG FCS medium and subsequent inoculation of two different cell types: the 549/D3 subclone, which is reported to be highly permissive for infection with the 47832c strain (1) and HepG2/C3A cells. A549/D3 were seeded the day before at a density of 1×10^4 cells/well in a 96well plate, HepG2/C3A at 2×10^4 cells/well. Both cell lines were already seeded in the respective media which were used for serial dilution. Seven days post infection immunofluorescence staining was performed followed by counting of FFU as stated in the HEV titration section.

HEV titration

To quantify the HEV infectivity titers, a serial dilution infection assay was performed using again both, HepG2 and HepG2/C3A as target cells, seeded the day before with 2×10^4 cells/well in either DMEM complete or MEM low IgG FCS medium. Virus was serially diluted threefold and used for inoculation of the respective target cells. Seven days later viral titers were determined by indirect immunofluorescence staining against the capsid and counting the number of focus forming units (FFU) containing ORF-2 positive cells. FFU were counted in three wells of highest dilutions where about 2-30 foci were detectable. One individual FFU is considered as one or

more ORF-2 positive cells were from another FFU by at least three negative cells. The average of the counted numbers of the respective FFU was calculated per milliliter. We further set the limit of quantification (LOQ) of respective titration assays to the lowest dilution with no cell death visible. The LOQ ranges between five and 15 FFU/mL, except for Fig. 5F, where cytotoxicity was increased (45 FFU/mL) due to ß-mercaptoethanol supplementation.

Infection of primary human and primary porcine hepatocytes

Virus stock of HEVcc intracellular p6_G1634R was produced as described in HepG2 in DMEM complete with freeze and thaw cycles in DMEM complete. The virus was filtered through a 0.45 µm filter to remove all potential leftover cell debris which might influence the quality of subsequent immunofluorescence staining. Afterwards virus was used for inoculation of the primary cells at an MOI of 1 and diluted – if necessary – in DMEM complete for the primary human hepatocytes (PHH) and in Williams´ medium E for the primary porcine hepatocytes (PPH). Human liver tissue for cell isolation was obtained from patients undergoing partial hepatectomy and after written informed consent was obtained. The protocol was approved by the local ethics commission (# 252-2008). Isolation of primary porcine hepatocytes was performed at the Laboratory for Animal Science of Hannover Medical School after approval by the Lower Saxony regional authority for consumer protection and food safety (Niedersächsisches Landesamt für Verbraucherschutz und Lebensmittelsicherheit (LAVES); 16-2374). The animals were kept under housing conditions of the EU-Guideline 2010/63 and valid animal regulation act (Tierschutz-Versuchstierverordnung des deutschen Tierschutzgesetzes). Calculation of MOI was performed following the titer determination in HepG2/C3A cells in MEM low IgG FCS medium. For the PHH standard assays, 4 h or 24 h *post* infection (p.i.) cells were washed three times with warm PBS and fresh MM250-1 (Lonza) or Williams´ medium E was added. For the PPH Williams´ medium E containing ß-mercaptoethanol as described above was added 24 h p.i. after the PBS washing. As a control 25 µM ribavirin was used in 1-well starting with the medium change. In the kinetic assay, ribavirin was already added to the control wells during infection for all time points. Medium was changed furthermore on day 3 or 4 and on day 5 p.i. for the PHH and on day 3 and 6 for the PPH. In the standard assays extra- and intracellular virus was harvested at day 7 p.i., in the kinetic assay at different time points, as described above. Freeze and thaw cycles for the intracellular virus were performed in MEM low IgG FCS medium in a five times lower volume as the harvested supernatant and the titer was determined on HepG2/C3A cells in MEM low IgG FCS medium. Cells for immunofluorescence were fixed at day 7 p.i.

Indirect immunofluorescence

If not stated otherwise cells were fixed at seven days post transfection or infection with 3% paraformaldehyde (PFA) in PBS and stained as described previously (3). Shortly, cells were stained for the ORF2-encoded capsid protein with an ORF2-specific rabbit hyperimmune serum (kindly provided by R. Ulrich, Friedrich Loeffler Institute, Germany) and using a goat anti-rabbit antibody (AlexaFluor 488 or 568 for the trans-complementation assay, Life Technologies, Darmstadt, Germany) as secondary antibody. DNA was labelled with DAPI (4′,6′-diamidino-2-phenylindole, Life Technologies). Images were taken with an Olympus IX81 inverted microscope with 10× or 40× objectives.

Quantification of ORF2 positive cells and cells per focus

Mean fluorescence intensities of ORF2 immunofluorescence were obtained from a 5-pixel wide cytoplasm ring (cytoring) following segmentation of DAPI stained nuclei using CellProfiler (4). To distinguish noninfected cells from infected cells a minimum intensity threshold was applied (Fig S3A, B). To determine the number of cells per focus, individual foci were masked by hand in FIJI (5) using ORF2 immunofluorescence images and DAPI stained nuclei were subsequently counted using CellProfiler.

Iodixanol density-gradient fractionation

Density gradient centrifugation was performed as recently described (6). In brief, samples were divided into 2×1 mL aliquots. Fractionation was performed by overnight centrifugation through an iodixanol step gradient (0 - 40%) at 154,000×g in a TH-641 swing-out rotor at 4°C using a Sorvall Ultra WX80 centrifuge. Thereafter, 10×1 mL fractions were collected and levels of viral RNA and viral infectivity of each fraction were determined. Buoyant densities were analyzed using a refractometer.

Trans-complementation

HepG2 cells were electroporated as described above using 5×10^6 HepG2 per electroporation and 5 µg HEV RNA of the p6 full length together with 5 µg of p6_G-Luc or p6_vGFP. After electroporation cells were resuspended in DMEM complete and seeded 2.5×10^6 cells per T25 flask. Four h p.t. medium was changed and Ribavirin was used at a final concentration of 25 µM as a control. Seven days p.t. intracellular virus was harvested following the same protocol as used for HEV produced in primary hepatocytes and the same target cell conditions for onward

infections. Virus titration of the co-electroporated p6_vGFP samples was performed and seven days later the titer of wild-type HEVcc containing the full length p6 genome was determined by staining the ORF2 protein in red followed by FFU counting versus GFP positive FFUs. For flow cytometry, 300,000 HepG2/C3A were seeded in 6-well dishes and inoculated with *trans*-complemented particles as described above. The undiluted intracellular virus of the co-electroporated p6_G-Luc samples was used to inoculate HepG2/C3A in a 96-well plate in duplicates or triplicates. Twenty-four h later cells were washed three times with PBS and fresh MEM low IgG FCS medium was added. Seven days p.i. the *Gaussia*-Luciferase activity was measured in the supernatant. For protection assay, RNase A solution was prepared with RNase A from bovine pancreas (Sigma-Aldrich, R6513) in a solution containing 10 mM Tris-HCl and 15 mM NaCl at pH=7.5. Aliquots were stored at -20 °C. For each experiment, a new vial was thawed. Virus stocks were incubated with RNase A at 37 °C for 60 minutes. The stocks were split either to immediately infect naïve cells or to perform RNA extraction for qPCR.

Gaussia luciferase measurement

Gaussia luciferase activity in supernatants of HEV83-2-27_Gluc transfected or HEVtcp infected cells was assessed at 4, 24, 48, and 72 hours *post* transfection (p.t.), 7 d p.i., respectively. Briefly, 20 µL of supernatant was transferred to a white, flat-bottom, 96-well microplate followed by the detection of luminescence using a microplate reader (Centro XS3 LB960; Berthold Technologies, Bad Wildbad, Germany) with coelenterazine as a substrate.

Flow cytometry

Cells were washed 3× with PBS, trypsinized and fixed in 3% PFA for 10 min at room temperature. After additional three PBS washes, cells were permeabilized for 20 min on ice with 0.1% Saponin in PBS with 1% horse serum (Gibco) and subsequently stained for the ORF2-encoded capsid protein with the described ORF2-specific rabbit hyperimmune serum (1/1200 in PBS with 5% horse serum) for 16 h at 4°C. Cells were washed twice with PBS with 5% horse serum and incubated with an α-rabbit mAb AF568 2^{nd}ary fluorescent dye (1/800) in PBS with 5% horse serum. Cells were stored on ice until analysis, which was performed with a MOFLO ASTRIOS EQ FACS sorter (Beckmann Coulter).

Quantitative real time PCR (qRT-PCR)

Cell Culture:

Viral RNA was extracted using the QIAamp Viral RNA Mini QIAcube Kit (Qiagen, Hilden, Germany). A QIAcube (classic) (Qiagen, Hilden, Germany) was used for automated sample preparation. Extracted RNA was stored at –80 °C until further analysis. For the SYBR Green based qRT-PCR, we used the GoTaq® 1-Step RT-qPCR System (Promega, Mannheim, Germany). The primer sequences for amplification of HEV RNA have been described previously (7): 5'-GGTGGTTTCTGGGGTGAC-3' (sense) and 5'-AGGGGTTGGTTGGATGAA-3' (antisense. A standard curve for the quantification of RNA copies was assessed by serial dilution of an RNA transcript. The transcript was generated by cloning the respective amplicon into the pCR 2.1-TOPO TA vector (ThermoFisher scientific, Waltham, USA) and transcribed into RNA by using the AmpliScribe™ T7 High Yield Transcription Kit (Epicenter, Madison, USA).

Trans-complementation:

As the above mentioned primer bind in HEV ORF3, which is replaced in the Gluc and vGFP reporter constructs used for *trans*-complementation, we here employed a previously published pair binding in ORF1 (8): 5′-ATTGGCCAGAAGTTGGTTTTCAC-3′ (sense) and 5′-CCGTGGCTATAATTGTGGTCT-3′ (antisense). A standard curve for the quantification of RNA copies was assessed by serial dilution of a HEV gt 3 Kernow-C1 p6 RNA transcript.

PHH:

Cellular RNA was isolated from lysed PHH at indicated time points p.i. using a Nucleo Spin RNA Kit (Macherey-Nagel, Düren, Germany) as described (9). Concentrations of the isolated RNA were quantified spectrophotometrically. Subsequently, 250 ng of total RNAs were reverse transcribed into cDNA using a PrimeScript First Strand cDNA Synthesis Kit (Takara Bio, Otsu, Japan). The primer sequences for amplification of HEV RNA targeting the ORF1 region are described above. For calculation of HEV RNA copy numbers, a standard curve was plotted for each run using a dilution of HEV gt 3 Kernow-C1 p6 plasmid DNA template

Iodixanol density-gradient fractionation:

For detection of cell-free viral RNA in gradient fractions, we used the QuantiTect Virus + ROC Viral Kit (Qiagen) and primer and probes as previously published (7) and described above.
All quantitative PCR were run on a LightCycler 480 system (Roche, Basel, Switzerland).

HEV fluorescent in situ hybridization (FISH)

Fixed HEV transfected cells were analyzed using the ViewRNA™ ISH Cell Assay Kit (Invitrogen, ThermoFisher Scientific) with specific Type 1 and Type 6 probe sets directed against the positive strand of HEV ORF1 and ORF2 sequences, respectively (10, 11). Fluorescent *in situ* hybridization (FISH) was performed according to the manufacturer´s protocol. Briefly, cells were pretreated with Detergent Solution QC® and Protease QS®. Following hybridization with target specific probe sets, pre-amplification and amplification steps, cells were hybridized with the Label probes. Nuclei were stained using DAPI (4′,6-diamidino-2-phenylindole). Images were acquired with an Olympus IX81 inverted microscope.

RNA-sequencing

Total RNA from primary cells of one representative PHH donor was extracted using a NucleoSpin RNA kit (Macherey-Nagel) according to the manufacturer's instructions. RNA quality was checked on an Agilent Bioanalyzer and sequencing libraries were generated using a NEBNext Ultra II Directional RNA Library Prep Kit (New England Biolabs) according to the manufacturer's instructions. Libraries were run on an Illumina NovaSeq 6000 platform. Data generated from individual samples (>30 million read per sample, paired-end read 50-mers) were mapped separately against the hg38 human reference genome. Gene expression was calculated for individual transcripts as reads per kilobase per million bases mapped (RPKM). All transcriptomic analyses were performed using CLC Genomics Workbench 11.0.1 (Qiagen, Aarhaus). All raw and mapped data discussed in this publication have been deposited in NCBI's Gene Expression Omnibus (12) and are accessible through GEO Series accession number GSE135619 (https://www.ncbi.nlm.nih.gov/geo/query/acc.cgi?acc=GSE135619).
Differentially expressed genes (DEGs) were identified by calculating fold changes in expression, p-values were corrected by taking false discovery rate (FDR) for multiple comparison into account.
Homo sapiens EBI Gene Ontology Annotation Database was used to execute Gene ontology (GO) Enrichment Analyses for biological processes. Gene identifiers for DEGs with absolute FC > 3, RPKM > 1 were used as input for identification of significantly enriched GO categories. P-values for specific GO categories were generated after Bonferroni correction for multiple testing.
Significant DEGs ($log_2FC > 1$, FDR < 0.05) were considered interferon regulated genes (IRGs) when listed in the CVR (Centre for Virus Research, University of Glasgow) "Orthologous

Clusters of Interferon-Stimulated Genes" database (settings: $\log_2$FC > 0 and FDR < 0.05; http://isg.data.cvr.ac.uk; (13)).

For generation of venn diagrams of overlapping DEGs, the package "venn" was used (14) implemented in the statistical programming language R (15).

Infection of HEVcc in liver-chimeric humanized mice

Homozygous uPA$^{+/+}$-SCID mice were transplanted with approximately 10^6 primary human hepatocytes (donor HH342, Corning, The Netherlands), as described previously (16). The registration number of the used animal facility at the Ghent University is LA1400070. To assess successful liver engraftment, the human albumin concentration in mouse plasma was determined by ELISA (Bethyl Laboratories, Montgomery, Texas, USA). Mice were then inoculated via intraperitoneal injection with cell culture produced HEV p6_wt (n=5, 2.5×10^4 FFU/mouse) or a variant thereof with the polymerase mutation p6_G1634R (n=4, 2.5×10^4 FFU/mouse). Fecal samples were collected on a weekly basis, whereas plasma samples were collected every two weeks. HEV RNA levels in mouse plasma and 10% (w/v) fecal suspensions were quantified using a previously described RT-qPCR protocol (17).

Statistical analysis

Differences in means of acquired data was tested for significance using GraphPad Prism v8.1.2 for Windows (La Jolla California USA, www.graphpad.com). For details regarding the statistical tests applied, please refer to the figure legends. P values < 0.05 were considered significant.

Supplementary Figures

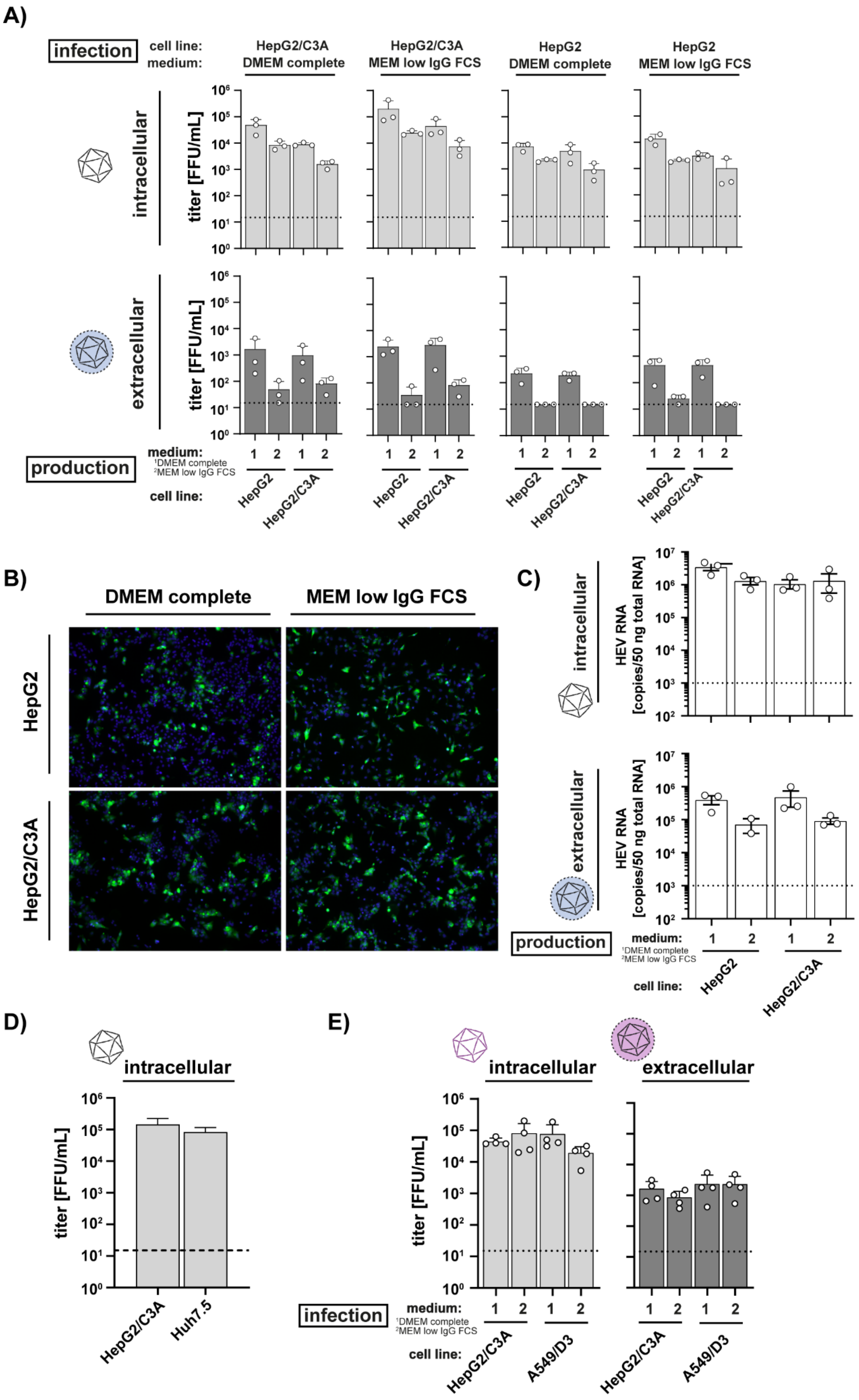

Figure S1: Optimizing cell culture conditions for high-titer virus production. A) Bar plots displaying mean titers of different combinations of cell lines and culture media used for the production of viral particles (x-axes, labelling below plots) and infection of target cells (x-axes, labelling above plots) for both intracellularly (upper panels) and extracellular (lower panels) particles. Titer is expressed as mean log of FFU (focus forming units) of three independent biological replicates (open circles, n = 3; mean ± SD; dashed line, limit of quantification (LOQ); titers below LOQ set to LOQ). **B)** Representative immunofluorescence images of HEV ORF2 positive cells after transfection of HEV p6_wt RNA in producer cells (HepG2, upper panel; HepG2/C3Al lower panel) cultivated in DMEM complete (left panel) or MEM low IgG FCS (right panel). Cell nuclei are shown in blue (DAPI) and HEV ORF2 positive cells in green (α-ORF2 pAb rabbit serum and α-rabbit mAb AF488 2ndary; 10× objective in widefield microscopy). All images were taken with individual automatic camera settings. Of importance, by visual examination, we noted lower fluorescence signal intensities in the green channel for in MEM low IgG FCS conditioned cells. **C)** HEV RNA copy numbers determined in lysate (intracellular, upper panel) and supernatant (extracellular, lower panel) of viral particle producing cell lines under different media conditions. RNA amount is expressed as mean copies per 50 ng of extracted total RNA of three independent biological replicates (open circles, n = 3; mean ± SD; for the second bar in the lower panel could not be analyzed; dashed line, limit of quantification). **D)** Mean titer of intracellular particles when inoculated on HepG2/C3A and Huh7.5 (n = 1; mean + SD of serial dilution titration; dashed line, limit of quantification (LOQ)). **E)** Bar plots displaying mean titers of HEV particles harvested from persistently infected A549 cells and infection of target cells (x-axes) for both intracellular (left panel) and extracellular (right panel) particles. Titer is expressed as mean log of FFU (focus forming units) of four independent biological replicates (open circles, n = 4; mean + SD; dashed line, limit of quantification (LOQ)).

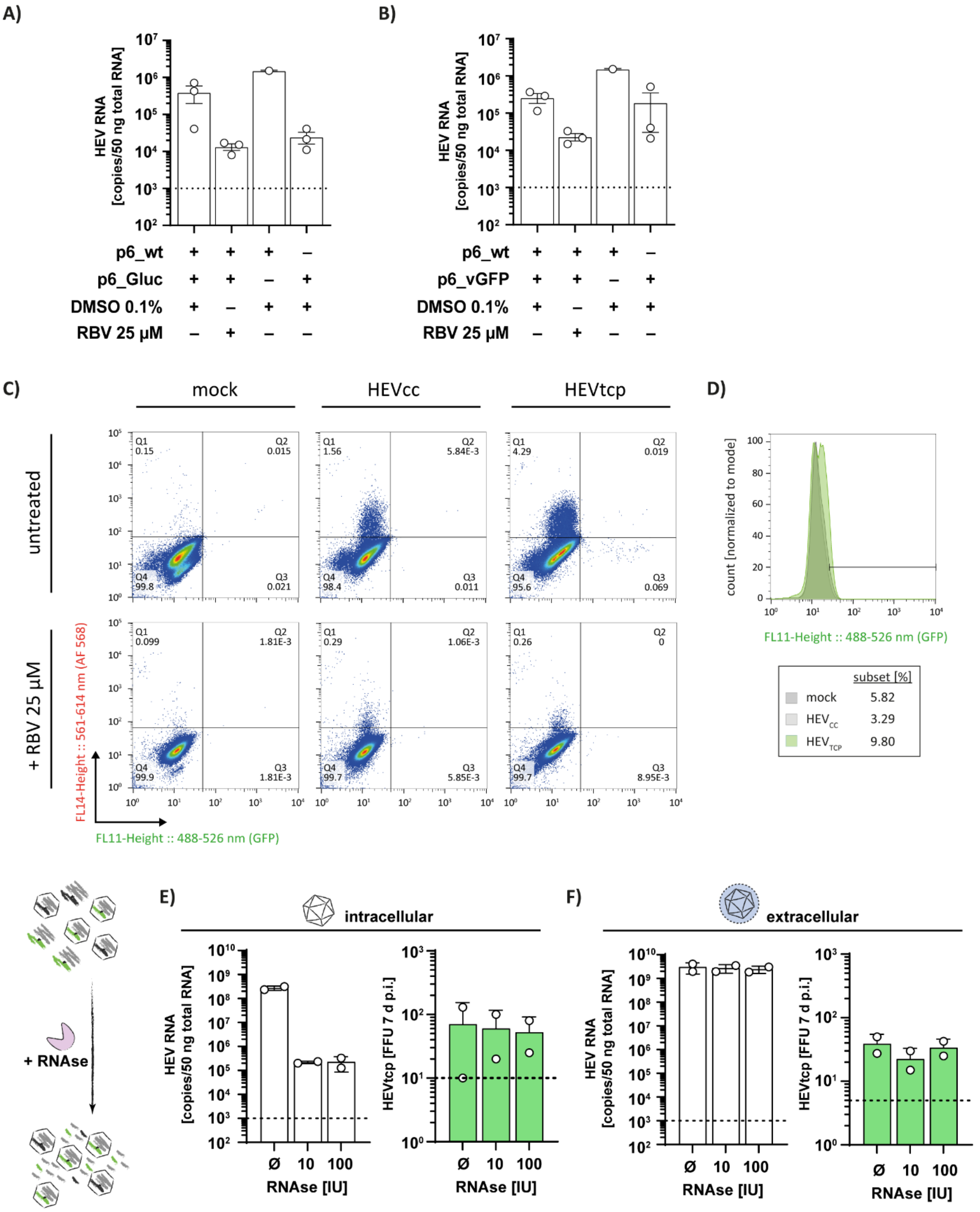

Figure S2: Production of infectious particles by *trans*-complementation of HEV reporter genomes. A) Bar plots showing qPCR results of intracellular particles harvested from producer cells transfected with full length p6_wt and subgenomic p6_Gluc and subsequently used for inoculation of target cells for flow cytometry. RNA amount is expressed as mean copies per 50 ng of extracted total RNA of three independent biological replicates (open circles, n = 3; mean ± SD; dashed line, limit of quantification). **B)** Bar plots showing qPCR results of intracellular

particles harvested from producer cells transfected with full length p6_wt and subgenomic p6_vGFP and subsequently used for inoculation of target cells for flow cytometry. RNA amount is expressed as mean copies per 50 ng of extracted total RNA of three independent biological replicates (open circles, n = 3; mean ± SD; for the third bar in both panels only one sample was available; identical virus stock of the full length HEV p6 was used in both setups, p6_Gluc and p6_vGFP, *ergo* same data points in both panels; dashed line, limit of quantification). **C)** Flow cytometric analysis of target cells inoculated with cell culture-derived (HEVcc, middle panels) and *trans*-complemented particles (HEVtcp, left panels). Cells were stained with α-ORF2 followed by AF568 (y-axis, Q1), representing full length particle infection events; GFP-positive (x-axis, Q3) cells were infected with *trans*-complemented reporter particles. Numbers in the plot indicate percentage of events in the respective quadrants. Virus-producing cells were either treated with 25 µM RBV (lower panels) or left untreated (upper panels); n =1. **D)** Histogram of normalized counts in GFP channel and percentage of area under the curve in the subset (horizontal bar). **E+F)** Bar chart representation of RNA protection assay. Intracellular **(E)** and extracellular **(F)** virions were treated with the indicated amount of RNase A for 60 min and residual HEV RNA copy numbers (clear bars) and infectivity (green bars) determined in two biological replicates (open circles, n = 2; mean ± SD; dashed line, limit of quantification).

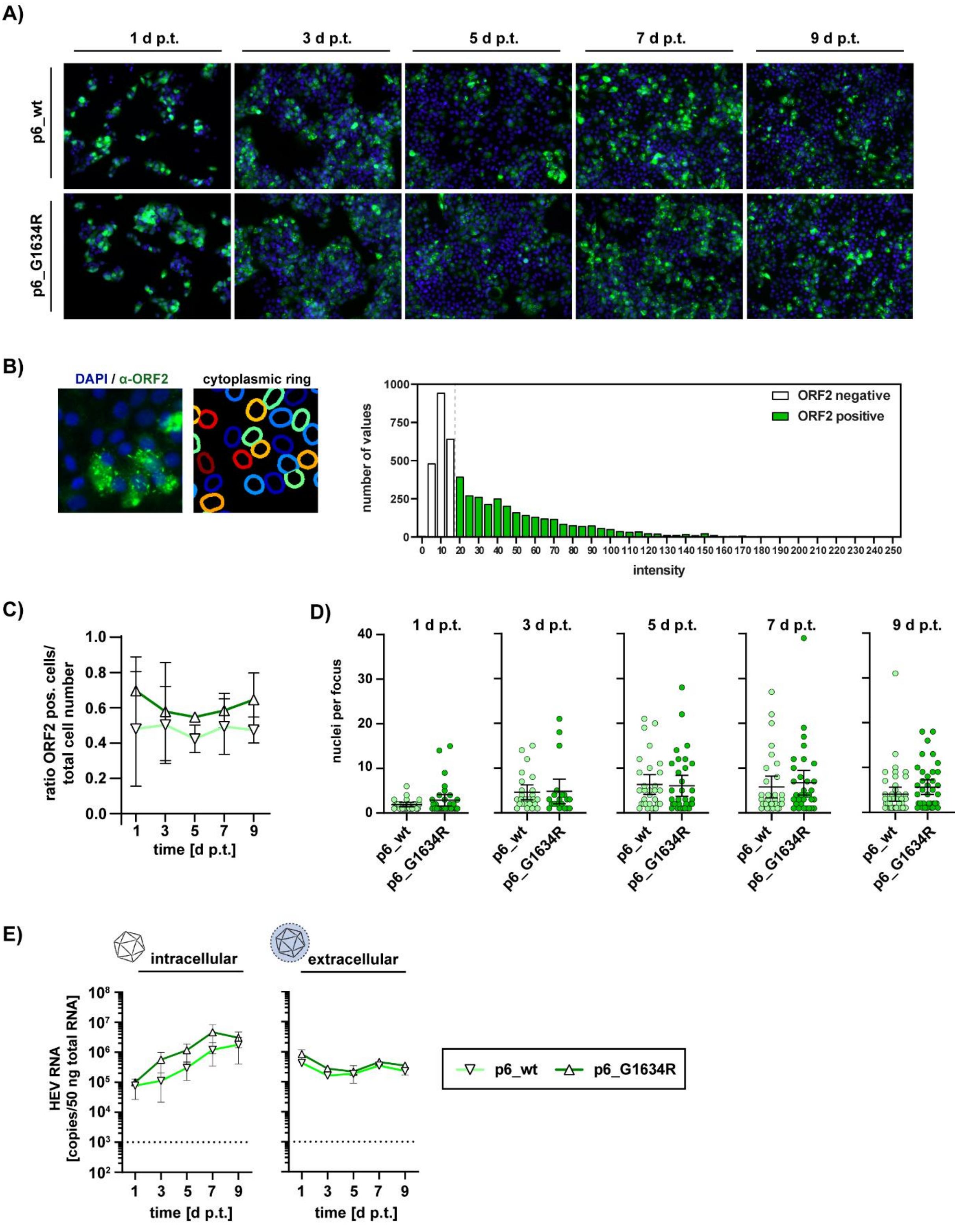

Figure S3: Introduction of the replication enhancing single nucleotide variant G1634R in HEVcc. A) Representative immunofluorescence images of HEV ORF2 positive foci after transfection of HepG2 cells with RNA of HEV p6_wt (upper panel) or p6_G1634R (lower panel) at different days post transfection (d p.t.). Cell nuclei are shown in blue (DAPI) and HEV ORF2 positive cells in green (α-ORF2 pAb rabbit serum and α-rabbit mAb AF488 2^{nd}ary; 20× objective

in widefield microscopy). **B)** Example of how HEV ORF2 positivity was assessed using cell profiler. Signal intensity was measured in a cytoplasmic, perinuclear ring and individual values binned in a histogram according to intensity (right panel, categorical x-axis). An arbitrary threshold (dashed line) distinguishes ORF2 negative (white bars) from positive (green bars) cells. **C)** Ratio of HEV ORF2 positive cells in p6_wt (light green line, inverted triangles) or p6_G1634 mutant (dark green line, triangles) HEV RNA transfected cultures monitored over time (n = 2 ± SD). **D)** Size of ORF2 positive foci in p6_wt (light green circle) or p6_G1634 mutant (dark green circle) HEV RNA transfected cultures monitored over time. The number of nuclei per focus were determined in one representative image in two independent experiments. **E)** QPCR results of intra- and extracellular particles harvested at indicated timepoints *post* transfection from producer cells HepG2. RNA amount is expressed as mean copies per 50 ng of extracted total RNA of three independent biological replicates (open circles, n = 2; mean ± SEM; dashed line, limit of quantification).

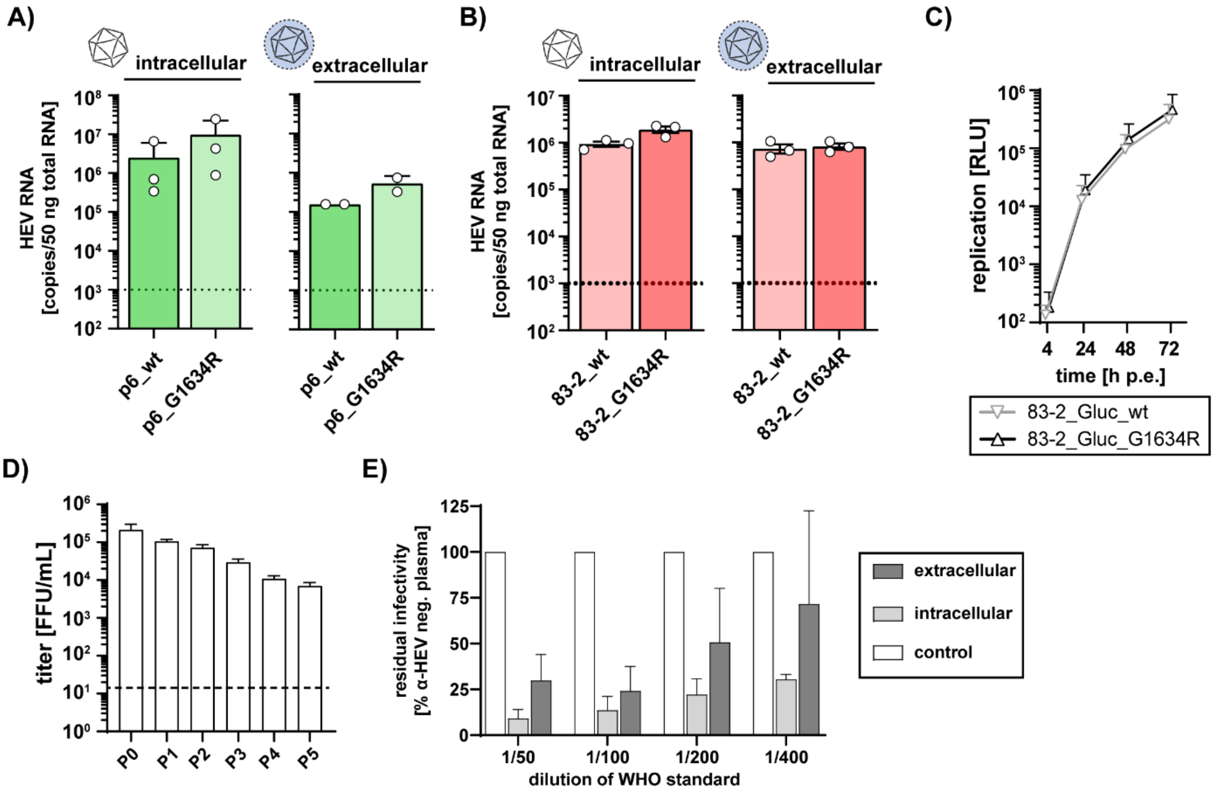

Figure S4: Characterization of HEVcc particles. A) Bar plots showing qPCR results of intra- and extracellular particles harvested from producer cells HepG2 transfected with p6_wt or p6_G1634R which were subsequently used for inoculation of target cells. RNA amount is expressed as mean copies per 50 ng of extracted total RNA of three independent biological replicates (open circles, n = 3; mean + SD; for the extracellular particles, only two sample were available; dashed line, limit of quantification). **B)** Bar plots showing qPCR results of intra- and extracellular particles harvested from producer cells HepG2 transfected with 83-2_wt or 83-2_G1634R which were subsequently used for inoculation of target cells. RNA amount is expressed as mean copies per 50 ng of extracted total RNA of three independent biological replicates (open circles, n = 3; mean + SD). **C)** Replication kinetics of HEV 83-2_Gluc_wt (gray line, inverted triangles) and HEV 83-2_Gluc_G1634R (black line, triangles) subgenomic RNA in transfected HepG2 cells. Replication was measured as relative light units using a luminometer (RLU, log-axis). n = 3 + SD; h p.e., hours post electroporation. **D)** Titers (FFU, focus forming units, log y-axis) of intracellular-derived HEV p6_G1634R particles produced in serially passaged HepG2 producer cells. Error bars indicate SD of titration assay. **E)** Neutralization of intracellular (light gray bars) and extracellular (dark gray bars) HEV p6_G1634R particles with a serially diluted WHO α-HEV polyclonal serum (18) (categorical x-axis). The residual infectivity was normalized to titers counted when using α-HEV negative serum (white bars combining data for intra- and extracellular; linear y-axis; n = 2 ± SD).

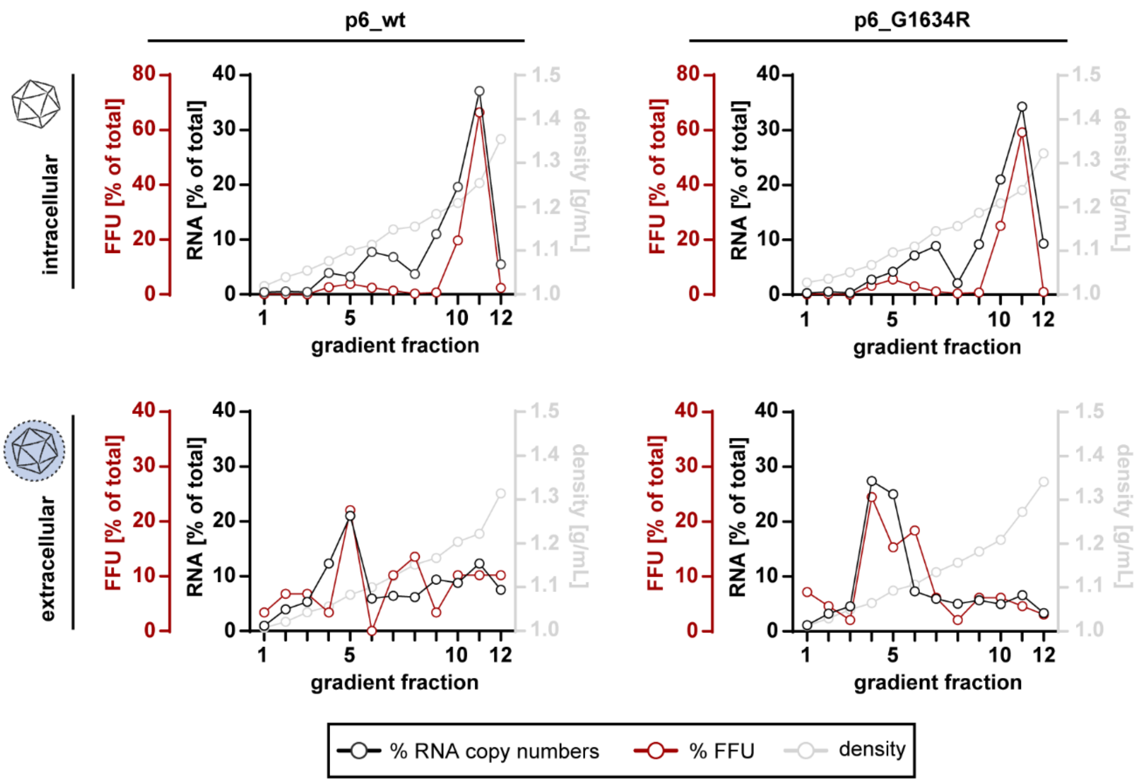

Figure S5: Biophysical characterization of HEV infectious particles. Density gradient fractions (x-axes) of isolated p6_wt (left panels) and p6_G1634R (right panels) viral particles harvested from lysed cells (intracellular, upper panels) and supernatant (extracellular, lower panels). Black lines and circles represent the number of RNA copies/mL (left black y-axes); titers measured in FFU/mL are depicted as red lines and circles (left red y-axes). The assessed density of the fractionated gradient is shown on the right y-axes in gray (representative plot of three biological replicates shown).

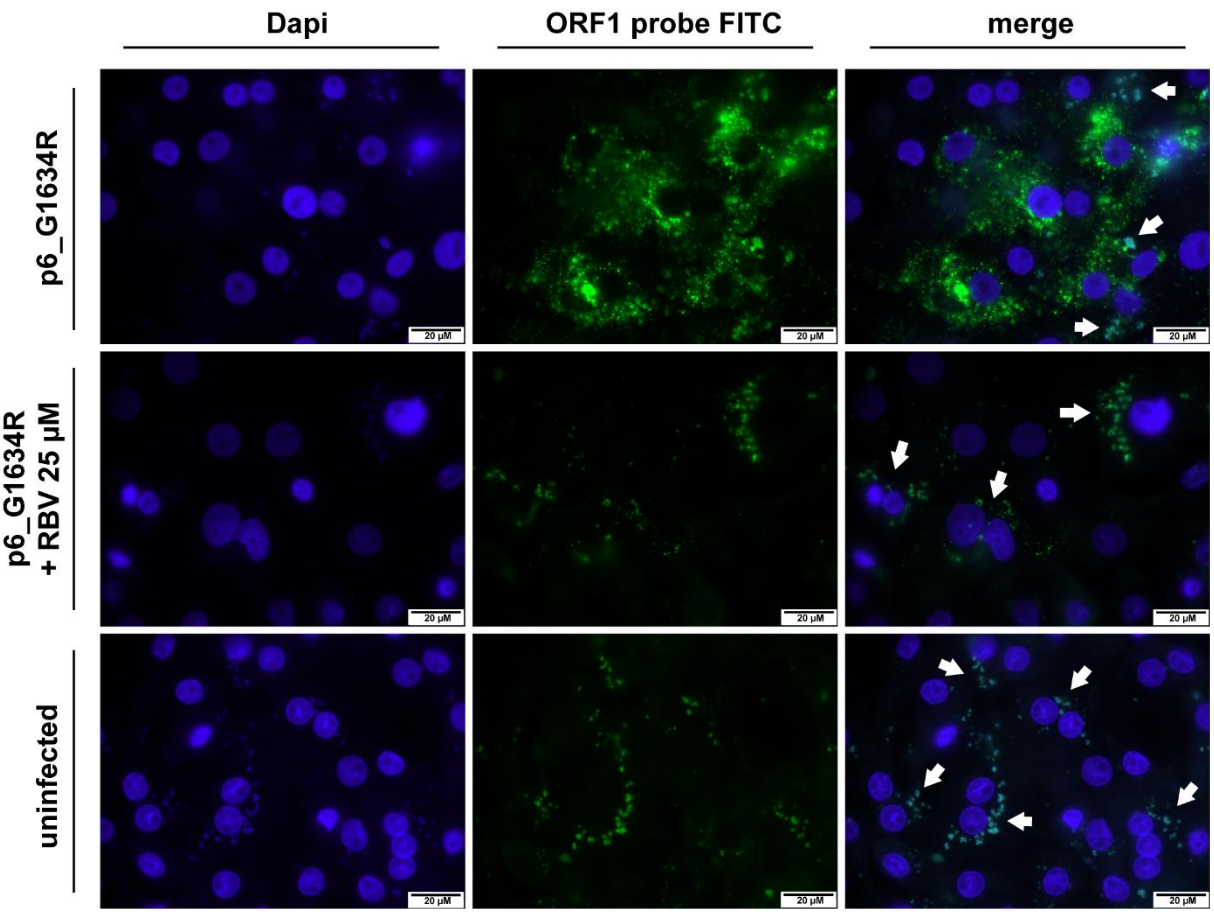

Figure S6: Fluorescence *in situ* hybridization (FISH) images of p6_G1634R infected primary human hepatocytes (PHH). Infected PHH were probed with complement labelled complement RNA targeting the HEV ORF1. Accumulation of ORF1 in the green channel in infected PHH indicate replication processes (upper panel). White arrows in all three merged images point to unspecific fluorescence signals, visible also in the Dapi channel and double-checked in the red channel (not shown).

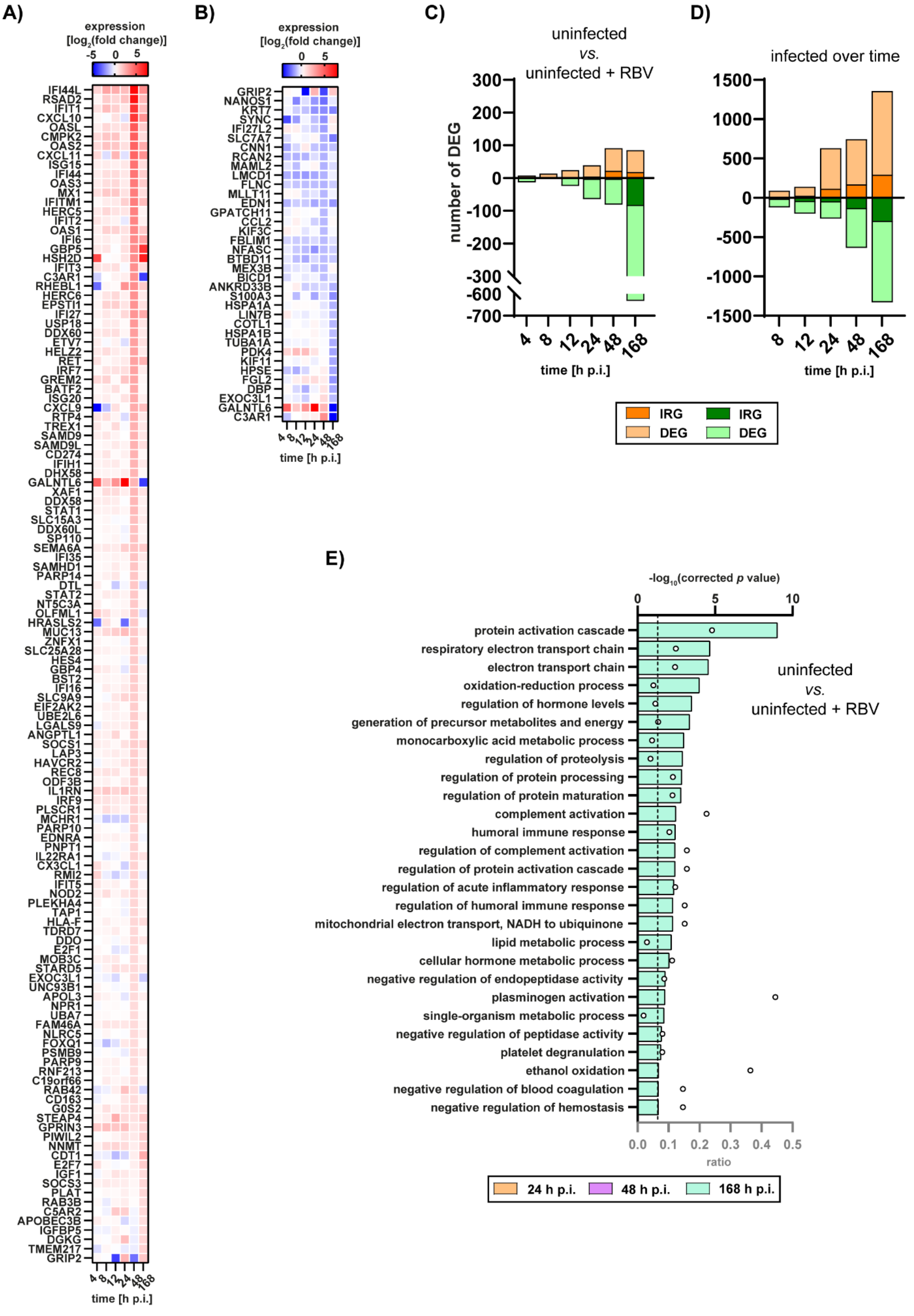

Figure S7: Transcriptional responses in PHH to HEVcc infection. A) Heatmap of all interferon regulated genes (IRG) significantly upregulated at least at one time point in HEVcc

infected PHH compared to uninfected cells (log_2FC), ordered by change in expression 48 h p.i. **B)** Heatmap of all IRG significantly downregulated at least at one time point in HEVcc infected PHH compared to uninfected cells (log_2FC), ordered by change in expression 48 h p.i. **C)** Total number of significant DEG (linear y-axes) up (light orange bars) or downregulated (light green bars) over time (categorical x-axes) in untreated mock infected compared to RBV 25 µM treated mock infected PHH. Fractions of previously described IRG are colored darker (h p.i., hours post infection). **D)** Total number of significant DEG (linear y-axes) up (light orange bars) or downregulated (light green bars) over time (categorical x-axes) in infected PHH normalized to 4 h p.i. Fractions of previously described interferon regulated genes (IRG) are colored darker. **E)** Representation of analysis of significant enriched (Bonferroni corrected p value < 0.05, dashed line, upper linear y-axis) pathways in untreated mock infected compared to RBV 25 µM treated mock infected PHH. Pathways are ordered according to significance with color of bars representing time point of enrichment (cyan 168 h p.i.). Open circles depict the number of regulated genes as ratio of the total number of genes assigned to the respective pathway (lower linear x-axis).

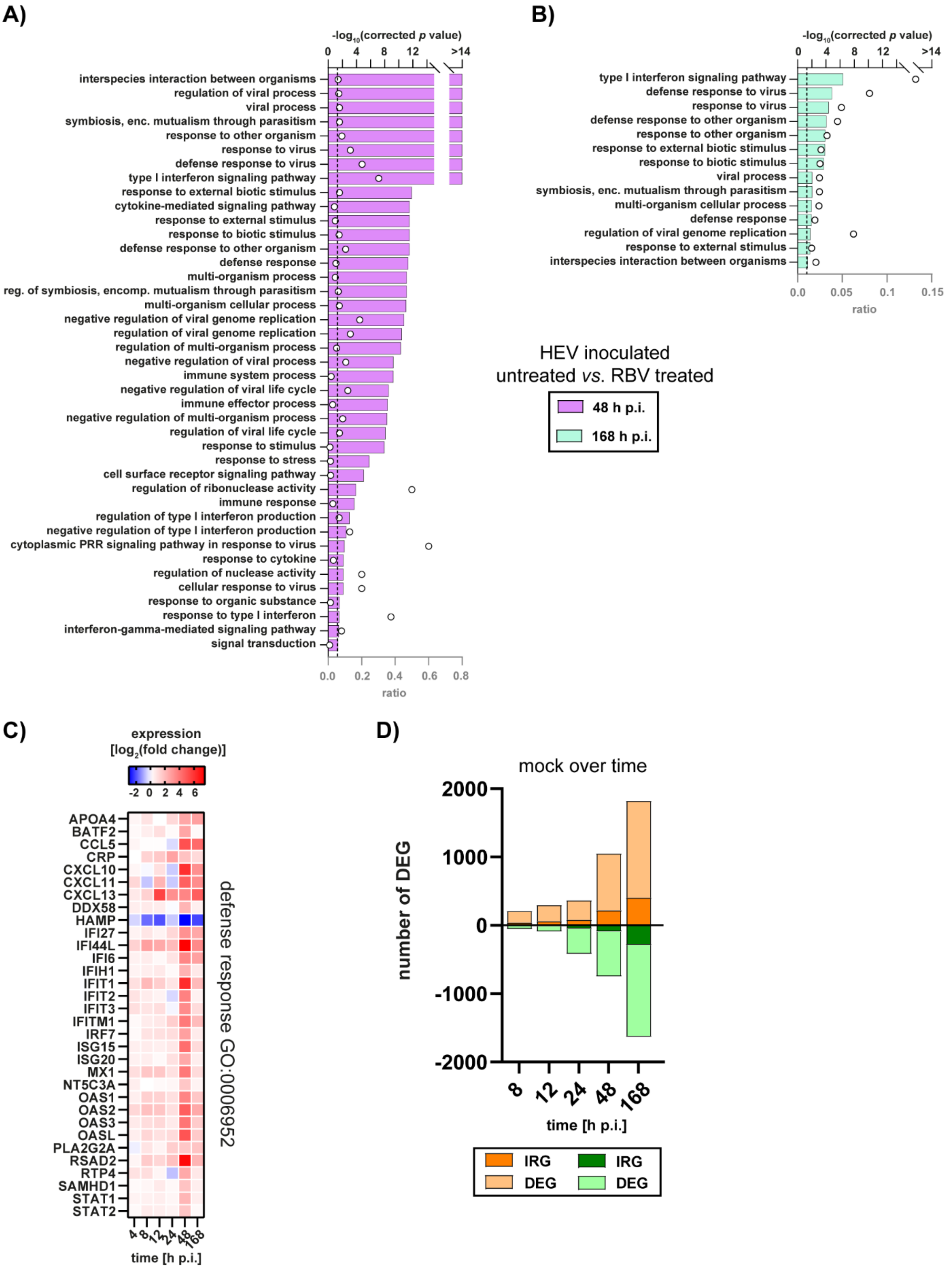

Figure S8: Transcriptional responses in PHH to HEVcc infection. A+B) Representation of analysis of significant enriched (Bonferroni corrected p value < 0.05, dashed line, upper linear y-axis) pathways in untreated HEVcc infected compared to RBV 25 µM treated HEVcc infected PHH. Pathways are ordered according to significance with color of bars representing time point

of enrichment (**A)** pink 48 h p.i.; **B)** cyan 168 h p.i.). Open circles depict the number of regulated genes as ratio of the total number of genes assigned to the respective pathway (lower linear x-axis). **C)** Heatmap of significant DEG in the pathway "defense response" (GO:0006952) in alphabetical order. Color code represents the fold change (FC) of the expression in HEVcc infected PHH compared to uninfected cells (log_2FC) at different time points post infection. **D)** Total number of significant DEG (linear y-axes) up (light orange bars) or downregulated (light green bars) over time (categorical x-axes) in untreated mock infected PHH compared to 4 h p.i. data set. Fractions of previously described interferon regulated genes (IRG) are colored darker (h p.i., hours post infection).

References

1. Schemmerer M *et al. (*2016) Enhanced Replication of Hepatitis E Virus Strain 47832c in an A549-Derived Subclonal Cell Line. *Viruses* 8.
2. Kleine M *et al. (*2014) Explanted diseased livers - a possible source of metabolic competent primary human hepatocytes. *PloS one* 9:e101386.
3. Todt D *et al. (*2018) The natural compound silvestrol inhibits hepatitis E virus (HEV) replication in vitro and in vivo. *Antiviral research* 157:151–158.
4. Kamentsky L *et al. (*2011) Improved structure, function and compatibility for CellProfiler: modular high-throughput image analysis software. *Bioinformatics (Oxford, England)* 27:1179–1180.
5. Schindelin J *et al. (*2012) Fiji. An open-source platform for biological-image analysis. *Nature methods* 9:676–682.
6. Bankwitz D *et al. (*2010) Hepatitis C virus hypervariable region 1 modulates receptor interactions, conceals the CD81 binding site, and protects conserved neutralizing epitopes. *Journal of virology* 84:5751–5763.
7. Jothikumar N, Cromeans TL, Robertson BH, Meng XJ, Hill VR (2006) A broadly reactive one-step real-time RT-PCR assay for rapid and sensitive detection of hepatitis E virus. *Journal of virological methods* 131:65–71.
8. Zhou X *et al. (*2014) Rapamycin and everolimus facilitate hepatitis E virus replication. Revealing a basal defense mechanism of PI3K-PKB-mTOR pathway. *Journal of hepatology* 61:746–754.
9. Knegendorf L *et al. (*2018) Hepatitis E virus replication and interferon responses in human placental cells. *Hepatology communications* 2:173–187.
10. Battich N, Stoeger T, Pelkmans L (2013) Image-based transcriptomics in thousands of single human cells at single-molecule resolution. *Nature methods* 10:1127–1133.
11. You X *et al. (*2015) Neural circular RNAs are derived from synaptic genes and regulated by development and plasticity. *Nature neuroscience* 18:603–610.
12. Edgar R, Domrachev M, Lash AE (2002) Gene Expression Omnibus: NCBI gene expression and hybridization array data repository. *Nucleic acids research* 30:207–210.
13. Shaw AE *et al. (*2017) Fundamental properties of the mammalian innate immune system revealed by multispecies comparison of type I interferon responses. *PLoS biology* 15:e2004086.
14. Dusa A (2018) *venn. Draw Venn Diagrams*. https://CRAN.R-project.org/package=venn.
15. R Core Team (2019) *R: A Language and Environment for Statistical Computing* (Vienna, Austria). https://www.R-project.org/.
16. Meuleman P *et al. (*2005) Morphological and biochemical characterization of a human liver in a uPA-SCID mouse chimera. *Hepatology (Baltimore, Md.)* 41:847–856.

17. Sayed IM *et al. (*2017) Study of hepatitis E virus infection of genotype 1 and 3 in mice with humanised liver. *Gut* 66:920–929.

18. Ferguson M, Walker D, Mast E, Fields H (2002) Report of a collaborative study to assess the suitability of a reference reagent for antibodies to hepatitis E virus. *Biologicals : journal of the International Association of Biological Standardization* 30:43–48.
